# Mangrove crab intestine and habitat sediment microbiomes cooperatively work on carbon and nitrogen cycling

**DOI:** 10.1371/journal.pone.0261654

**Published:** 2021-12-31

**Authors:** Prasert Tongununui, Yuki Kuriya, Masahiro Murata, Hideki Sawada, Michihiro Araki, Mika Nomura, Katsuji Morioka, Tomoaki Ichie, Kou Ikejima, Kohsuke Adachi

**Affiliations:** 1 Department of Marine Science and Environment, Faculty of Science and Fisheries Technology, Rajamangala University of Technology Srivijaya, Tambon Maifad, Amphur Sikao, Trang, Thailand; 2 Graduate School of Medicine, Kyoto University, Kyoto, Kyoto, Japan; 3 Maizuru Fisheries Research Station, Field Science Education and Research Center, Kyoto University, Maizuru, Kyoto, Japan; 4 National Institutes of Biomedical Innovation, Health and Nutrition, Tokyo, Japan; 5 Faculty of Agriculture, Kagawa University, Miki-cho, Kita-gun, Kagawa, Japan; 6 Faculty of Agriculture and Marine Science, Kochi University, Nankoku, Kochi, Japan; KAUST University, SAUDI ARABIA

## Abstract

Mangrove ecosystems, where litter and organic components are degraded and converted into detrital materials, support rich coastal fisheries resources. Sesarmid (Grapsidae) crabs, which feed on mangrove litter, play a crucial role in material flow in carbon-rich and nitrogen-limited mangrove ecosystems; however, the process of assimilation and conversion into detritus has not been well studied. In this study, we performed microbiome analyses of intestinal bacteria from three species of mangrove crab and five sediment positions in the mud lobster mounds, including the crab burrow wall, to study the interactive roles of crabs and sediment in metabolism. Metagenome analysis revealed species-dependent intestinal profiles, especially in *Neosarmatium smithi*, while the sediment microbiome was similar in all positions, albeit with some regional dependency. The microbiome profiles of crab intestines and sediments were significantly different in the MDS analysis based on OTU similarity; however, 579 OTUs (about 70% of reads in the crab intestinal microbiome) were identical between the intestinal and sediment bacteria. In the phenotype prediction, cellulose degradation was observed in the crab intestine. Cellulase activity was detected in both crab intestine and sediment. This could be mainly ascribed to *Demequinaceae*, which was predominantly found in the crab intestines and burrow walls. Nitrogen fixation was also enriched in both the crab intestines and sediments, and was supported by the nitrogenase assay. Similar to earlier reports, sulfur-related families were highly enriched in the sediment, presumably degrading organic compounds as terminal electron acceptors under anaerobic conditions. These results suggest that mangrove crabs and habitat sediment both contribute to carbon and nitrogen cycling in the mangrove ecosystem via these two key reactions.

## 1. Introduction

Mangroves are highly productive intertidal ecosystems that can transfer organic matter and nutrients from forest sediments to the coastal ocean [1–7]. It has long been considered that mangrove leaf litter is a major carbon source that is degraded in the sediment and converted into detrital materials as the basis of the detritus food chain in coastal areas [1, 4, 5]. Moreover, it is generally recognized that mangrove sediment is bioturbated by macrofauna and microfauna, and sesarmid mangrove crabs are the main bioturbating organisms in mangrove ecosystems [8–10]. Sesarmid crabs feed on massive litterfalls and release organic matter as feces into the soil burrow, which can retain as much as 80% of the primary production of mangrove ecosystems [5, 11–15]. Several studies suggest that the sesarmid crab burrows, which presumably comprise components partly derived from crab intestinal systems, can have an impact on metabolism in the sediment [11, 12, 16–18]. In addition, the abundance and diversity of crabs can affect organic matter degradation and sediment characteristics[17, 19–21].

There are several reports about microbiomes in mangrove sediment for a better understanding of its roles in sulfur, nitrogen, and carbon metabolism [22–25]. Recent studies have reported that non-foraging fiddler crabs in mangroves can modulate the microbiome of the mangrove environment [26–28], where the fiddler crabs can alter the characteristics of the sediment via symbiotic bacteria and their burrowing behavior. In addition to the mangrove ecosystem, the relationship between symbiotic bacteria and habitats/nests using metagenomics to analyze microbe-animal associations has been reported for termites [29], birds [30, 31], and earthworms [32]. However, limited information is available about the effect of the intestinal bacteria of leaf-eating sesarmid crabs on the mangrove sediment microbiome and their associated metabolic effects on carbon and nitrogen.

Cellulose, a main component of the litter, is a linear, undegradable homopolymer consisting of glucose units linked by β-1,4 bonds [33]. Cellulase is a general term for cellulose-degrading enzymes, which can be classified into three types based on the mode of enzymatic action and substrate specificity: endoglucanases (EC 3.2.1.4), exoglucanases (EC 3.2.1.74 and 3.2.1.91), and β-glucosidases (EC 3.2.1.21) [34]. In mangrove sediments, several microbes and genes encoding enzymes have been identified to be involved in cellulose degradation, which can promote litter degradation [35]. In addition, endogenous enzymes in the digestive organs have been identified in a wide variety of invertebrates [34, 36] including mangrove crabs [37–39]. Analysis of sesarmid mangrove crabs has shown that mangrove litter fragments dominate (often over 90%) in stomach contents, and stable isotope technique analysis supported that leaf litter is the main carbon source for sesarmid crabs [11, 40, 41]. On the other hand, the assimilation efficiency of litter by crabs is not high, and the remaining fragmented matter is released as feces [42–44]. Thus, mangrove crabs first shred the litter to increase the surface area and chemically degrade the cellulose in the stomach to assimilate it; however, limited information is available on the cellulolytic activity of crab intestinal bacteria before defecation and further microbial degradation in the sediment.

In contrast to the dominance in diet and importance as a carbon source, mangrove litter is considered a poor nitrogen source with a carbon to nitrogen (C/N) ratio of more than 100 [43]. There has been a long-term debate on how sesarmid crabs obtain the necessary nitrogen, where food selectivity of supplementary N-rich diets has been the main topic [11]. Recent studies using stable isotope analysis suggest that the dominant nitrogen sources for crabs are animal tissues and microphytobenthos (MPB) [11, 41, 45]. However, information on nitrogen metabolism in mangrove crabs is more limited than that on carbon metabolism [46–48]. Nitrogen fixation is a key reaction for the nitrogen cycle in ecosystems because it requires high energy because of the cleavage of the triple bond in N_2_. In mangrove ecosystems, nitrogenase has been detected in sediments and the rhizosphere [49–53]. Recently, a significant contribution of N_2_ fixation by fiddler crab carapace-associated biofilm to mangrove nitrogen cycling has been suggested [26]. Furthermore, our preliminary examination showed significant N_2_ fixation activity by sesarmid crab intestinal bacteria, supporting the importance of benthic animal (crab)-microbe associations (holobionts) in the mangrove nitrogen cycle.

The purpose of this study was to investigate the microbiomes of intestinal bacteria and habitat sediments of sesarmid crabs in mangroves to understand their metabolic functions in carbon and nitrogen cycling. To examine the links between crab intestines and sediment regions, we determined five distinct areas of mangrove sediment, including the sesarmid crab nest burrow, and investigated the intestinal microbiomes of three species of sesarmid crabs (*Neosarmatium smithi*, *Episesarma versicolor*, and *Perisesarma indiarum*). After predicting the metabolic function of the samples, we performed key enzymatic assays (cellulase and nitrogenase) to validate the possible cooperative roles of sediments and crabs in the material flow of the mangrove ecosystem.

## 2. Materials and methods

### 2.1. Samples

**Fig 1:**
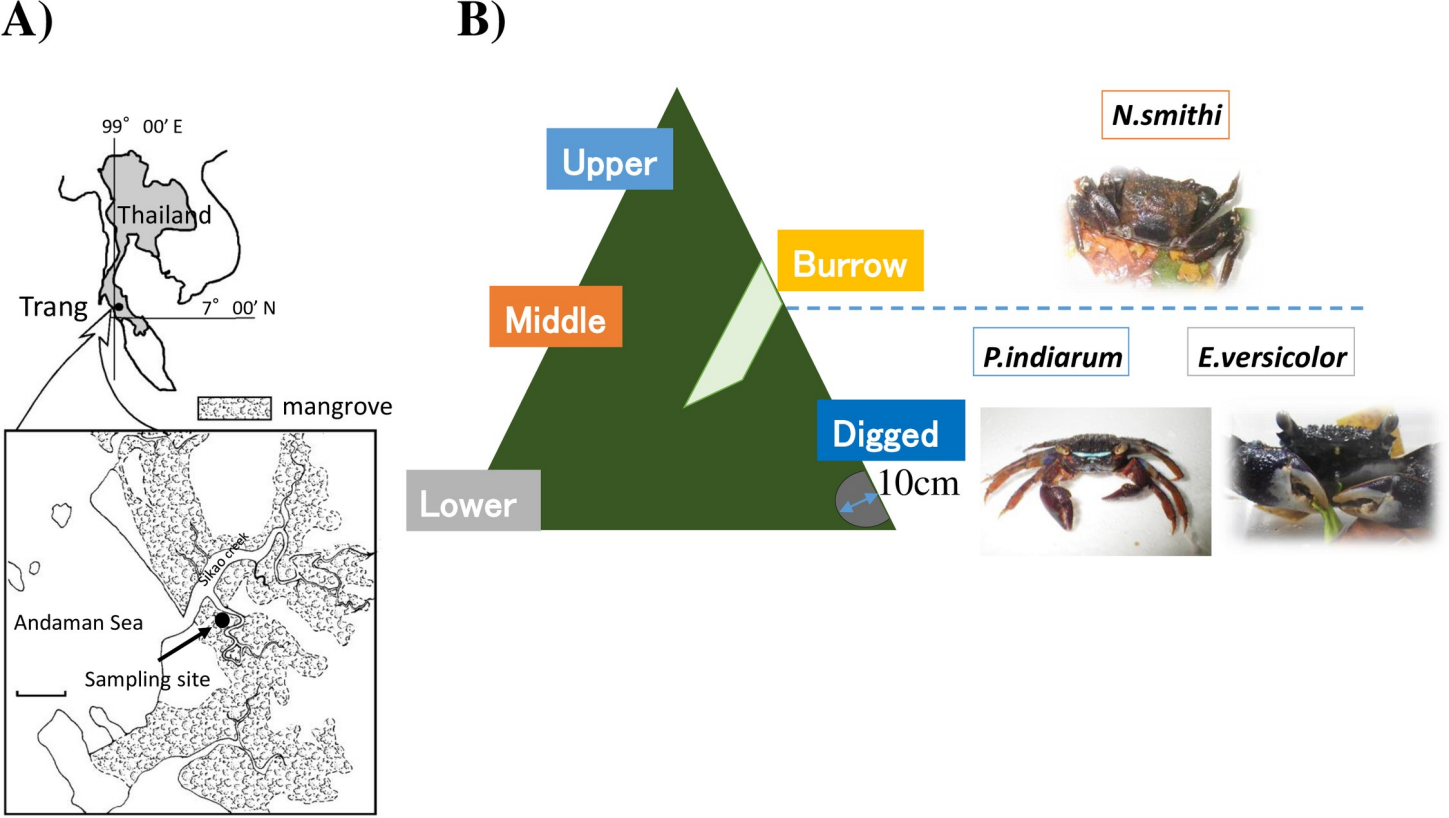
Location of experimental station and sampling points. A) The map of Rajamangala University of Technology Srivijaya, Amphur Sikao. B) Model of the sediment samples. There were four mounds at the station for sediment collection with a height of approximately 100 cm each. Sediment samples were collected from five regions: upper (U1-4), middle (M1-5), lower (L1-5), burrow (B1-3), and dug regions (D1-3). The upper region is completely immersed only during high tide in spring. The lower region is regularly flooded twice a day. *N*. *smithi* (NS1-4) were normally found in the middle region of the mound, whereas *N*. *versicolor* (NV1-4) and *P*. *indiarum* (PI1-4) were found in the lower region.

Sediments in and around lobster mounds were collected in the mangrove forest located on the campus of Rajamangala University of Technology, Srivijaya (Trang, Thailand, 7°53N, 99°31E) on December 26, 2013 and 18–22th December 2014 (Fig 1A), which were new quarter moon and full moon periods, respectively. The forest was dominated by *Rhizophora apiculata* mangrove trees and mud lobster (*Thalassina anomala*) occurring in abundance. We performed a preliminary transect survey (30 m × 5 transects), recording three microhabitat types found in the forest floor (lower open space, dense cover of prop roots, and lobster mound), and found that 31% of the forest floor area at the study site was covered by lobster mounds. The mound heights ranged from 80 to 140 cm, with a basal diameter ranging from 100 to 250 cm, and were completely immersed during high tide during the full moon (spring tide). Sesarmid crabs, *N*. *smithi*, *E*. *versicolor*, and *P*. *indiarum*, dig burrows in and around the mounds. Four lobster mounds were set as the sampling site, and sediments were collected from the: 1) upper, 2) middle, and 3) lower (flat and open space) regions of the mud lobster mound, 4) crab burrow, and 5) sediment dug out at a depth of 10 cm from the mound (Fig 1B). Individuals of the sesarmid crab species *N*. *smithi*, *E*. *versicolor*, *and P*. *indiarum* were sampled from a mangrove forest located at the same site in December 2014 during low tide (Fig 1B). The crabs were identified based on their morphologies. The intestinal contents (feces) were collected immediately after sampling and frozen at -80°C until use for metagenomic analysis. The samples used were identical to those described above for the cellulase assay. For the nitrogenase assay, samples were collected in 17th November 2017 (last quarter moon), which was vacuum-packed immediately after sampling, transferred to a temperature of 4°C, and measured 72 h after sampling. Sample collection did not require permission because the mangrove forests, particularly those part of the study site, are openly accessible for harvesting aquatic resources, and the crabs are often captured by locals.

### 2.2. Metagenome analysis

#### 2.2.1. DNA extraction

DNA of the sediment bacteria was isolated using NucleoSpin Soil (TaKaRa, Kyoto, Japan) following the manufacturer’s instructions. The sample (250 mg) was first lysed with SL1 and Enhancer SX buffer, horizontally vortexed for 5 min at RT, and centrifuged (11,000 × g, 1 min) to obtain the supernatant containing DNA. The residue was again dissolved in SL2 buffer and vortexed to obtain the supernatant. Genomic DNA was isolated from mixed solutions. The intestinal contents of bacterial DNA were isolated using the same methods, except for a lower amount of starting material (100 mg).

#### 2.2.2. PCR amplification

The protocol for metagenome analysis followed that reported by Kim (2013) [54]. The forward primer 27Fmod (5’-AGRGTTTGATYMTGGCTCAG-3’) and reverse primer 338R (5’-TGCTGCCTCCCGTAGGAGT-3’) containing 10 bp multiplex identifiers and the Roche GS junior adaptors were used to amplify the V1-V2 region of the 16S rRNA gene from the metagenomic DNA using Astec PC-320. The PCR mixture contained 0.5 U of Extaq (TaKaRa), 2.5 μL of reaction buffer, 2.5 μL of dNTPs (10 mM), 0.2 μM of each primer, and 4 ng of gel-purified genomic DNA, and the total volume was adjusted to 25 μL with double distilled water. The cycling conditions were as follows: initial denaturation at 98°C for 1 min, followed by 20 cycles at 95°C for 15 s, 55°C for 30 s, and 72°C for 60 s, followed by a final 2-min extension at 72°C. The reaction was performed in triplicate for each sample. The products were checked by electrophoresis on a 1% (w/v) agarose gel. The DNA amplicons were gel-purified using Agencourt AMPure XP beads (Beckman Coulter, Brea, CA, USA) following the manufacturer’s instructions, and the concentrations were determined using an Agilent BioAnalyzer 2100 (Invitrogen, Waltham, MA, USA) and a Nanodrop spectrophotometer (Thermo Scientific, Waltham, MA, USA). The triplicate amplicons for the same sample were pooled together at equimolar ratios before sequencing.

#### 2.2.3. Pyrosequencing of the 16S rRNA gene amplicons

Amplicons were combined in a single tube at equimolar concentrations. The pooled amplicon mixture was purified twice (AMPure XP kit, Agencourt, Takeley, United Kingdom), and the cleaned pool was purified using the PicoGreen assay (Thermo Scientific, Waltham, MA, USA). The pool was then diluted in TE to 10^5^ molecules/mL, and 30 μL of this pool was added to the emulsion PCR reaction to attain a ratio of 0.3 molecules of amplicon per bead. Pyrosequencing was performed using a 454 Life Sciences GS Junior (Roche, Basel, Switzerland).

#### 2.2.4. Sequence data analysis

Sequence filtration was performed by evaluating sequence lengths larger than 200 bp and matching the barcode sequences of 16S rRNA pyrosequences obtained from the samples. Reads were assigned to relevant samples based on barcode sequences prior to the data analysis. Sequence data analysis was performed using the Quantitative Insight Into Microbial Ecology (QIIME) package [55]. This package enables operational taxonomic unit (OTU)-based community identification (97% similarity), picking representative sequences, taxonomic assignment using the RDP classifier [56], and construction of a phylogenetic tree using the FastTree method. The analysis results were imported to a developed pyrosequencing workflow program (OTUMAMi), which automatically computes the individual read counts and cumulative counts per sample. Microbial community statistics based on different levels of phylogeny were also computed. Taxonomic grouping of operational taxonomic units with read counts greater than nine in total was extracted as the major microbial community. Then, the reads were merged according to their phylogenetic affiliation, and the community compositions were interpreted with representative sequences. A phylogenetic tree was constructed using the same set of sequences using the QIIME. Sequence data from this study have been deposited in the DDBJ DRA database (https://ddbj.nig.ac.jp/DRASearch) under accession numbers DRR299057-DRR299088.

#### 2.2.5. Data processing

The rarefaction curves and related parameters were calculated using EstimateS. The abundance of OTUs was square-root transformed to calculate similarity. Generalized linear models with negative binomial families were used to assess the differential abundances of OTUs for crabs and sediment with the ’DESeq2’ package within R [57, 58]. The differences in OTUs among each sample were statistically analyzed with a Wald test with the Benjamini-Hochberg method to correct *p*-values for false discovery rate at alpha values of 0.1. Non-metric multidimensional scaling (nMDS) was conducted for sediment and crab intestinal bacteria based on the dissimilarity among OTUs assessed by Bray-Curtis similarities, followed by the analysis of similarity (ANOSIM) to test for significant differences between the bacterial communities. The nMDS and ANOSIM were performed using the PRIMER v5 software package (PRIMER-E Ltd, Ivy Bridge, UK). Phenotype analysis was performed using METAGENassist and PICRUST2 MetaCyc (over 1% frequency on average) [59, 60]. The assigned OTUs were statistically analyzed using the Wald test with the Benjamini–Hochberg method to correct *p*-values for false discovery rate at alpha values of 0.1.

### 2.3. Cellulase assay

Cellulase activity in the sediment and intestinal contents was determined following the method of Deng (1994) [61], with slight modifications. Briefly, samples (50 mg) were placed in a 1.5 mL tube and incubated with 25 μL toluene and 1 mL of 2% carboxymethyl cellulose (CMC) (Sigma-Aldrich, St Louis, USA) in 50mM acetate buffer (pH 5.5) at 30°C for 24 h. After mixing, the samples were centrifuged three times. To remove minerals, the supernatants were transferred into 1.5 mL tubes, treated with K-saturated cation exchange resin (200 mg) (DOWEX 500Wx8 Cation Exchange Resin, Wako, Osaka, Japan), shaken for 30 min, and the supernatants were analyzed for reducing sugars using the Somogyi-Nelson method with glucose as a standard (Wako, Osaka, Japan). Two controls were included in the study. One was buffered 2% CMC incubated with toluene but without sample (intestinal contents or sediment), and the other was a sediment sample incubated with toluene and acetate buffer (Wako, Osaka, Japan) without CMC. After incubation, the values of the two controls were subtracted from the glucose values obtained for the CMC-treated samples.

### 2.4. Acetylene reduction assay (nitrogenase assay)

Nitrogenase activity was determined by acetylene reduction activity (ARA), in which ethylene was used as the standard. The intestinal contents were collected from the rectum of the crabs and transferred to a vacuum pack at 4°C. In the laboratory, each sample was placed in a 25-mL vial, gassed with argon, and incubated at 37°C with 2.6 mL acetylene. After 30 min, ethylene formation was measured by gas chromatography using a Shimadzu GC-8A gas chromatograph (Shimadzu, Kyoto, Japan) [62]. ARA is fragile and the assay is time-consuming; therefore, the analysis focused on the intestines of *N*. *smithi and E*. *versicolor* and the sediments of the upper, burrow, and root regions. Root sediment was collected from the root region of *Rhizophora apiculata* in the same field.

### 2.5. Stable isotope analysis

All samples were placed in tin capsules after oven-drying for 48 h at 60°C and ground to a fine powder using a ball mill (MM200, Retsch, Haan, Germany). The δ^15^N and δ^13^C values of these samples were measured using an isotope mass ratio spectrometer (Delta V Advantage; Thermo Fisher Scientific, Waltham, MA, USA) connected to an elemental analyzer. The natural abundance of ^15^N or ^13^C is expressed in per mil (‰) deviation from international standards using the following equation: δ^15^N or δ^13^C  =  (R_sample_/R_standard_ − 1), where R_sample_ and R_standard_ are the isotopic ratios (^15^N/^14^N or ^13^C/^12^C) of the sample and standard, respectively. The standards were atmospheric nitrogen and Vienna Pee Dee belemnite for nitrogen and carbon, respectively. We used multiple working standards calibrated to international standards (IAEA-N1, IAEA-N2, and IAEA-NO-3 for δ^15^N; NBS19, L-SVEC, and IAEA-CH6 for δ^13^C). The SD of repeated measurements of multiple working standards was <0.2‰ for both isotope ratios. This analysis focused on *N*. *smithi* and *E*. *versicolor*.

## 3. Results

### 3.1. Metagenome analysis of intestinal bacteria in mangrove crabs

In total, 21630 reads were obtained from rarefaction analysis and Chao richness estimates predicted the presence of over 1422 bacterial OTUs (Table 1 and S1 and S2 Files; 97% confidence interval range: 1372–1471 OTUs). The Chao 1 index was 419±318, 476±259, and 352±78 for *N smithi*, *E*. *versicolor*, and *P*. *indiarum*, respectively, and the Shannon diversity index showed that *E*. *versicolor* and *P*. *indiarum* were similar, while the Simpson index varied among individuals due to the variance of homogeneity in these two crabs (Table 1). In *N*. *smithi*, the Simpson index was similar between individual crabs, indicating that the homogeneity of OTU was unchanged among the samples. In the Venn diagram analysis of crabs, 105 out of the 1422 OTUs were shared by the three crab species, while 400, 362, and 289 OTUs were unique to *N smithi*, *E*. *versicolor*, *and P*. *indiarum*, respectively (S3 File). A number of shared OTUs related to *Rhodobacteraceae* (16 OTUs), *Marinilabiaceae* (15 OTUs), and *Demequinaceae* (5 OTUs) were identified (S1 File).

**Table 1:** Parameters for metagenome analysis.

species	sample name	year	reads	Coverage	OUT	Chao1	Shannon	Simpson Inv
*N*. *simthi*	NS1	2014	2335	93.05	217	347.48	3.82	19.52
	NS2	2014	5521	74.92	250	439.41	3.8	23.62
	NS3	2014	1231	90.68	156	247.96	3.89	25.12
	NS4	2014	1121	97.22	163	333.37	3.82	18.75
*N*. *versicolor*	EV1	2014	3539	93.33	475	837.97	4.43	23.14
	EV2	2014	610	90.35	253	494.85	4.98	71.83
	EV3	2014	526	71.62	93	183.29	3.24	8.82
	EV4	2014	1657	88.49	113	160.02	3.45	18.24
*P*. *indiarum*	PI1	2014	1875	96.19	236	485.87	3.6	10.06
	PI2	2014	2177	97.94	408	829.83	4.94	60.52
	PI3	2014	370	94.23	170	371.68	4.74	74.73
	PI4	2014	582	92.77	124	219.97	3.82	22.45
region	sample name	year	reads	Coverage	OUT	Chao1	Shannon	Simpson Inv
Upper	U1	2013	16384	93.41	2401	3707.33	6.33	159.12
	U2	2013	5332	88.41	1262	2009.52	6.14	167.03
	U3	2014	1823	89.19	390	747.32	5.04	73.3
	U4	2014	1892	63.21	1008	2464.22	6.5	338.47
Middle	M1	2013	10287	86.18	3723	6749.46	7.52	672.29
	M2	2013	6527	78.72	2447	4524.19	7.19	609.06
	M3	2013	12535	85.78	3561	5767.88	7.34	543.74
	M4	2014	730	49.18	491	6856.04	5.99	286.51
	M5	2014	5552	74.05	2338	4931.33	7.19	586.44
Lower	L1	2013	10015	78.56	3801	1442.96	7.6	808.98
	L2	2013	6139	73.17	2827	1696.11	7.51	952.73
	L3	2013	16016	86.18	4055	3635.77	6.78	93.57
	L4	2014	972	52.06	635	4971.4	6.23	365.35
	L5	2014	1690	69.64	791	1785.32	6.17	219.87
Nest	N1	2013	31539	91.77	5497	9009.2	7.36	551.49
	N2	2014	1241	57.53	729	1883.08	6.25	300.86
	N3	2014	3364	72.15	1493	3147.29	6.8	457.97
Dgged	D1	2013	19448	88.87	4297	7160.58	7.17	339.54
	D2	2014	4612	82.37	1388	2708.03	6.29	192.32
	D3	2014	3178	66.55	1606	7082.98	6.94	516.08

**Fig 2:**
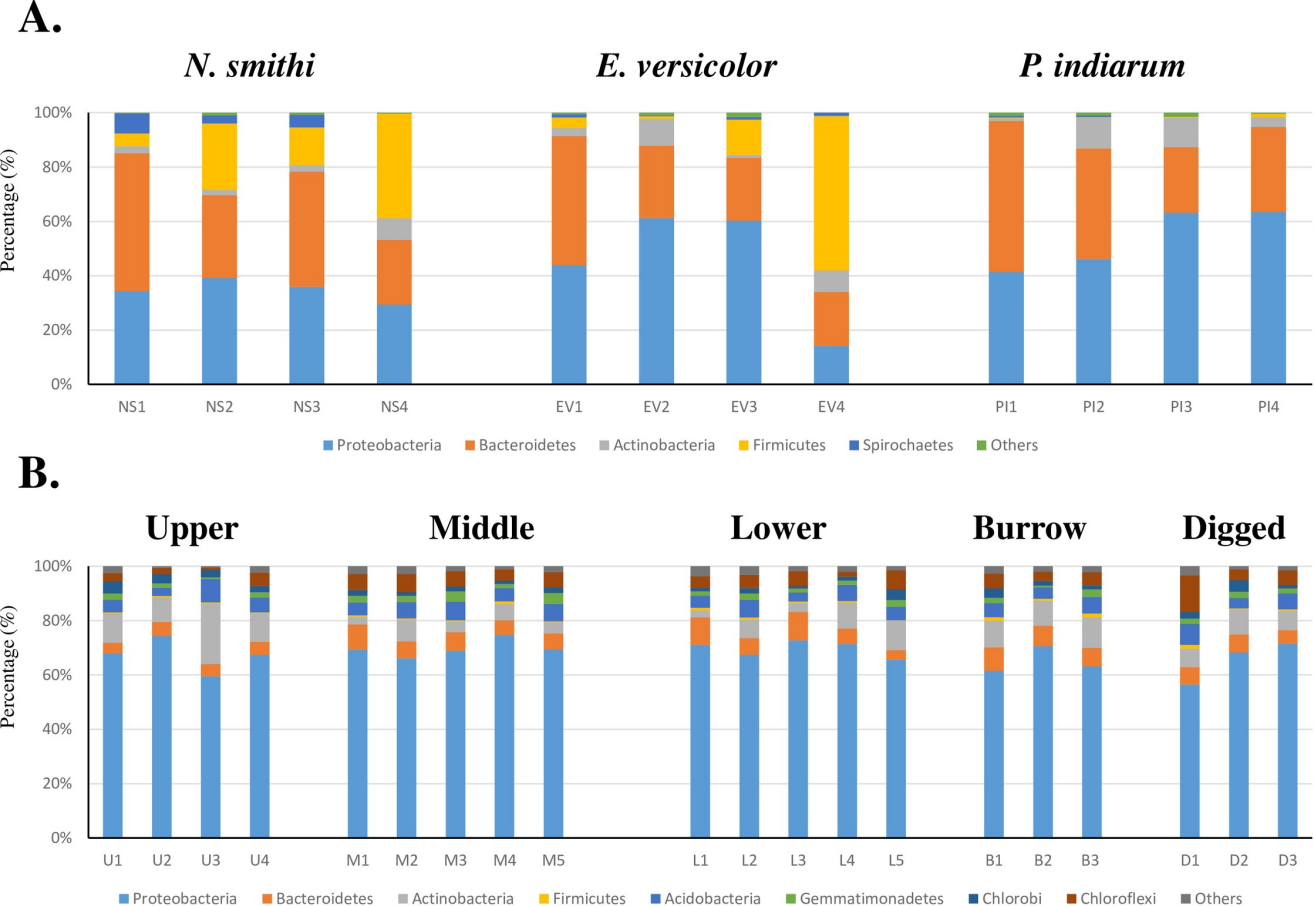
Phylum distribution with relative abundance. Tags are classified using a threshold of 97%. The bar indicates the relative abundance in each sample. A) Crab intestines. B) Sediment regions.

At the phylum level of the microbial flora, 89% of the OTUs were assigned on average, regardless of the species. Proteobacteria (44.3±15.5%; Mean±SD), Bacteroidetes (34.6±12.25%), Firmicutes (13.0±17.8%), and Actinobacteria (5.3±4.0%) were dominant in the intestines of mangrove crabs regardless of the species (Fig 2A and S4 and S5 Files). One exception was one *E*. *versicolor* individual (EV4) that was collected from the sandier substrate. It showed a low percentage of Proteobacteria (13.9%) and a high percentage of Firmicutes (55.5%). After removing the data of EV4 from the statistical analysis, the levels of Firmicutes of *N*. *smithi* (20.2±14.3%) *and E*. *versicolor* (18.3±25.3%) were significantly higher than those of *P*. *indiarum* (0.48±0.52%) based on the Wald test (alpha<0.1). At the family level (S4 and S5 Files), 70.1% of the OTUs were assigned on average. *Rhodobacteraceae*, *Marinilabiaceae*, *Pseudoalteromonadaceae*, *Vibrionaceae*, *Demequinaceae*, and *Shewanellaceae* were present in all species at over 2% on average. For Wald test (alpha<0.1) in *N*. *smithi*, *Marinilabiaceae* (38.9±11.4%), *Spirochaetaceae* (4.2±3.2%), *Demequinaceae* (3.5±2.0%), and *Oceanospirillaceae* (2.6%±2.9%) were present at a significantly higher level compared with their level in the other two species. The percentage of *Lachnospiraceae* (19.5±16.9% in *N*. *smithi* and 16.2±26.9% in *E*. *versicolor*) was significantly higher than that *in P*. *indiarum* (0.38±0.48%).

### 3.2. MDS analysis of intestinal bacteria in mangrove crabs

**Fig 3:**
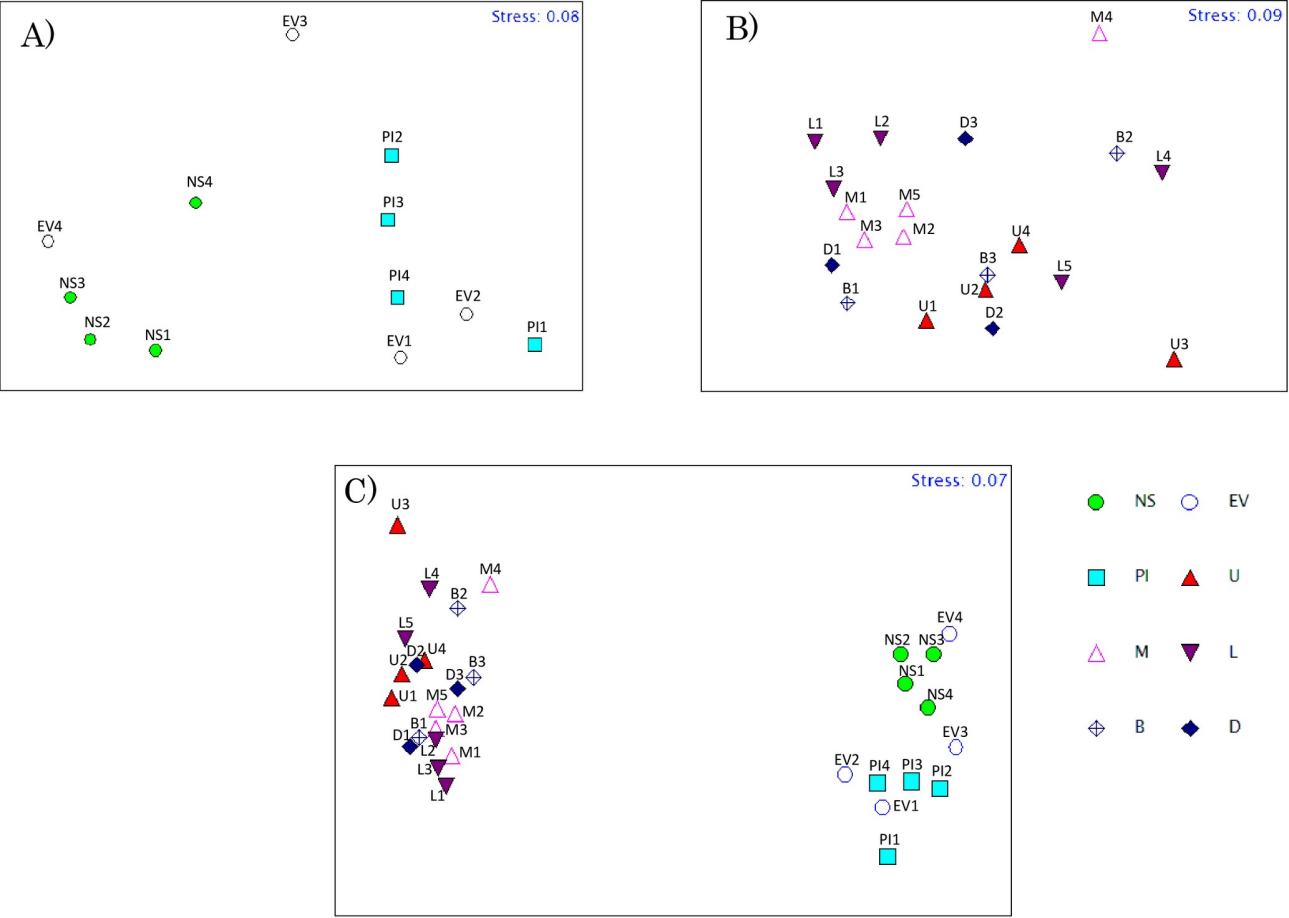
MDS analysis of the microbiome profiles of crab intestines and sediments. A) Crab intestines. B) Sediment regions. C) The combined graph of crab intestines and sediments. The symbols NS, EV, PI indicate the profiles of *N*. *smithi*, *E*. *versicolor*, and *P*. *indiarum* intestine, whereas U, M, L, B, and D denote the upper, middle, lower, burrow, and dug region, respectively. In ANOSIM analysis (permutations = 9999), the value for sample statistic (Global R) and significance level of sample statistic were (0.343 and 0.041), (0.025 and 0.369), and (0.529 and 0.001) for A), B), and C) respectively.

In the MDS analysis using OTU data (Fig 3A), the samples in *N*. *smithi* were more closely grouped, whereas those of *E*. *versicolor and P*. *indiarum* were more scattered in distinct areas without species-specific clustering. *E*. *versicolor* individual 4 (EV4) was an exception for the analyses, as reflected in the data in Fig 2A and S4 and S5 Files. The ANOSIM test revealed significant differences among the crab species (*p* <0.05).

### 3.3. Phenotype prediction of intestinal bacteria in mangrove crabs

**Fig 4:**
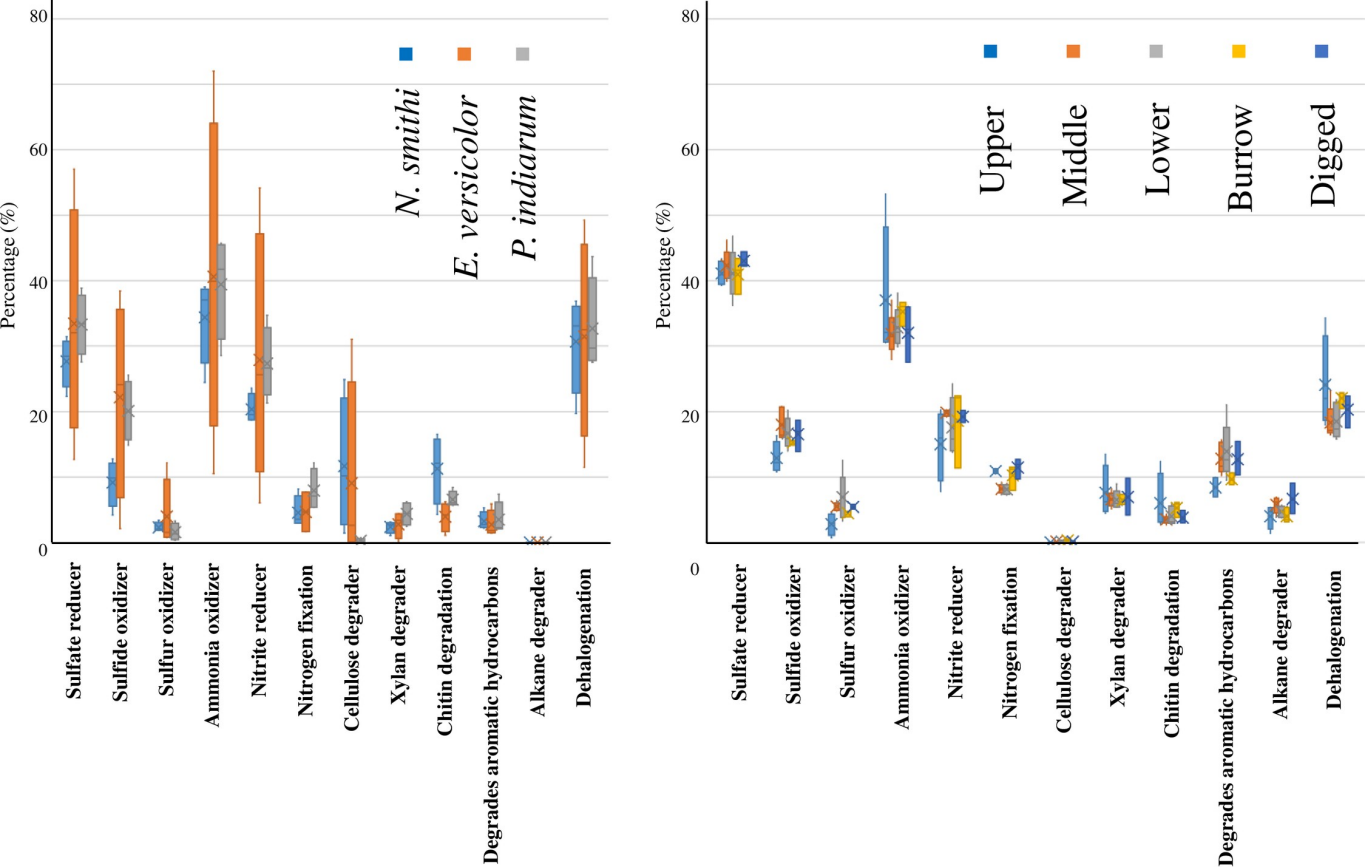
Phenotype prediction of sediment region and intestinal bacteria in mangrove crabs. The Y axes represent the percentage of OTUs assigned in METAGENassist, whereas the X axes represent the phenotype. Each bar represents the data for samples. A) Crab intestines. B) Sediment regions.

We analyzed the phenotype of each sample using the MTGAGENassist. In the metabolic phenotype analysis, a significant number of OTUs were enriched in nitrogen cycle-related phenotypes (ammonia oxidizer, nitrite reducer, nitrogen fixation), regardless of the species (Fig 4A). Carbon-related metabolism processes, such as degradation of aromatic carbons, xylan degradation, chitin degradation, and cellulose degradation, were enriched in all species except for cellulose degradation in *P*. *indiarum* (Fig 4A). It should be noted that *Demequinaceae* reads were not assigned to these phenotypes as discussed further. Very few reads (less than 1%) were mapped to the lignin degrader, regardless of the species (not shown). Sulfur-related phenotypes (sulfur reducer sulfide oxidizer and sulfur oxidizer) were also enriched in the sediment, as described later. In the Wald test (alpha<0.1), levels of cellulose degraders and sulfide oxidizers in *N*. *smithi* were significantly higher than those in other species. These data were supported by PICRUSt2 prediction including nitrogen fixation (S6 File).

### 3.4. Metagenome analysis of the sediment

In total, 159276 reads were obtained in the analysis of the amplicons sampled in 2013 and 2014 (Table 1 and S1 File). Rarefaction analysis and Chao richness estimates predicted the presence of over 19036 bacterial OTUs in total (97% confidence interval range: 18875–19196 OTUs) (Table 1 and S2 File). In the Venn diagram analysis, only 873 OTUs out of the 19036 identified OTUs were shared among the five regions, while 803, 3128, 4648, 2087, and 1613 were unique to the upper mound, middle mound, lower mound, burrow, and dug regions, respectively (S3 File). On average, 86% of the OTUs were assigned to a phylum. Regardless of the samples, Proteobacteria was the most dominant (67.6±4.8%), followed by Actinobacteria (8.1±4.3%) and Bacteroides (6.4±1.9%) (Fig 2). At the family level (S4 and S5 Files), 43.3% of the OTUs were assigned on average. Among them, the dominant profiles looked similar, but there was some regional dependency. *Desulfobacteraceae* was the most dominant (16.2±5.0%), irrespective of the samples, followed by *Desulfobulbaceae* (10.2±5.4%) and *Piscirickettsiaceae* (5.6% ±4.5%). In the upper part of the mound, the profile varied depending on the samples, but the most dominant family was *Desulfobacteraceae* (11.6±5.7%), followed by *Hyphomicrobiaceae* (11.3±3.7%), *Mariprofundaceae* (8.5±4.4%), and *Rhodobacteraceae* (8.1±3.8%), where the latter three were significantly higher than those of the other sampled regions in the Wald test (2013; alpha<0.1). The profile for the middle mound, lower mound, and burrow were similar, whereby *Desulfobacteraceae* (18.6±1.8, 16.1±2.9, and 12.8±2.9%, respectively) and *Desulfobulbaceae* (14.3±4.1, 8.9±3.7, and 11.0±6.9%, respectively) were dominant, followed by *Piscirickettsiaceae* (8.8±2.0, 10.4±4.4, and 5.1±3.9%, respectively) and *Hyphomicrobiaceae* (5.3±1.3, 4.8±2.1, and 6.1±1.7%, respectively) (S5 File). It is noteworthy that *Demequinaceae*, which was rarely observed in other regions, was enriched in the two samples in the burrow (1.6% and 1.3% in B1 and B3, respectively; S4 and S5 Files), although no statistical difference was observed in the Wald test.

### 3.5. MDS analysis of the sediment bacteria

In the MDS analysis for 2013 (Fig 3B), upper (U1 and U2), middle (M1, M2, and M3), and lower (L1, L2, and L3) mound regions were positioned closely, suggesting a regional similarity between the profiles in the same year. Although the results in 2014 did not follow this manner, some data from the same site sampled in 2013/2014, such as M5 and M1-3, were present in close space. However, ANOSIM did not show a significant difference between sediment samples (Global R = 0.025, P = 0.369; Fig 3C), possibly due to fluctuations between the two years. When the sediment and crab intestine data were collated in the same graph, the cluster of the crab intestine samples was clearly distinct from that of sediment bacteria samples, and ANOSIM revealed significant differences among the samples (Global R = 0.343, P = 0.041; Fig 3C).

### 3.6. Phenotype prediction of the sediment bacteria

In the metabolic phenotype analysis (Fig 4B), a significant number (up to 40%) of OTUs were enriched in sulfur (sulfur reducer, sulfide oxidizer, and sulfur oxidizer) and nitrogen (ammonia oxidizer, nitrite reducer, and nitrogen reducer) cycle-related phenotypes. Carbon-related metabolism, such as degradation of aromatic carbons, alkane degraders, xylan degradation, and chitin degradation, were enriched. The percentage of cellulose degraders and lignin degraders was less than 1%, regardless of the region. No clear statistical regional difference was observed in the Wald test. These data were supported by PICRUSt2 prediction including nitrogen fixation.

### 3.7. Venn diagram analysis of crab intestine and sediment OTUs

**Fig 5:**
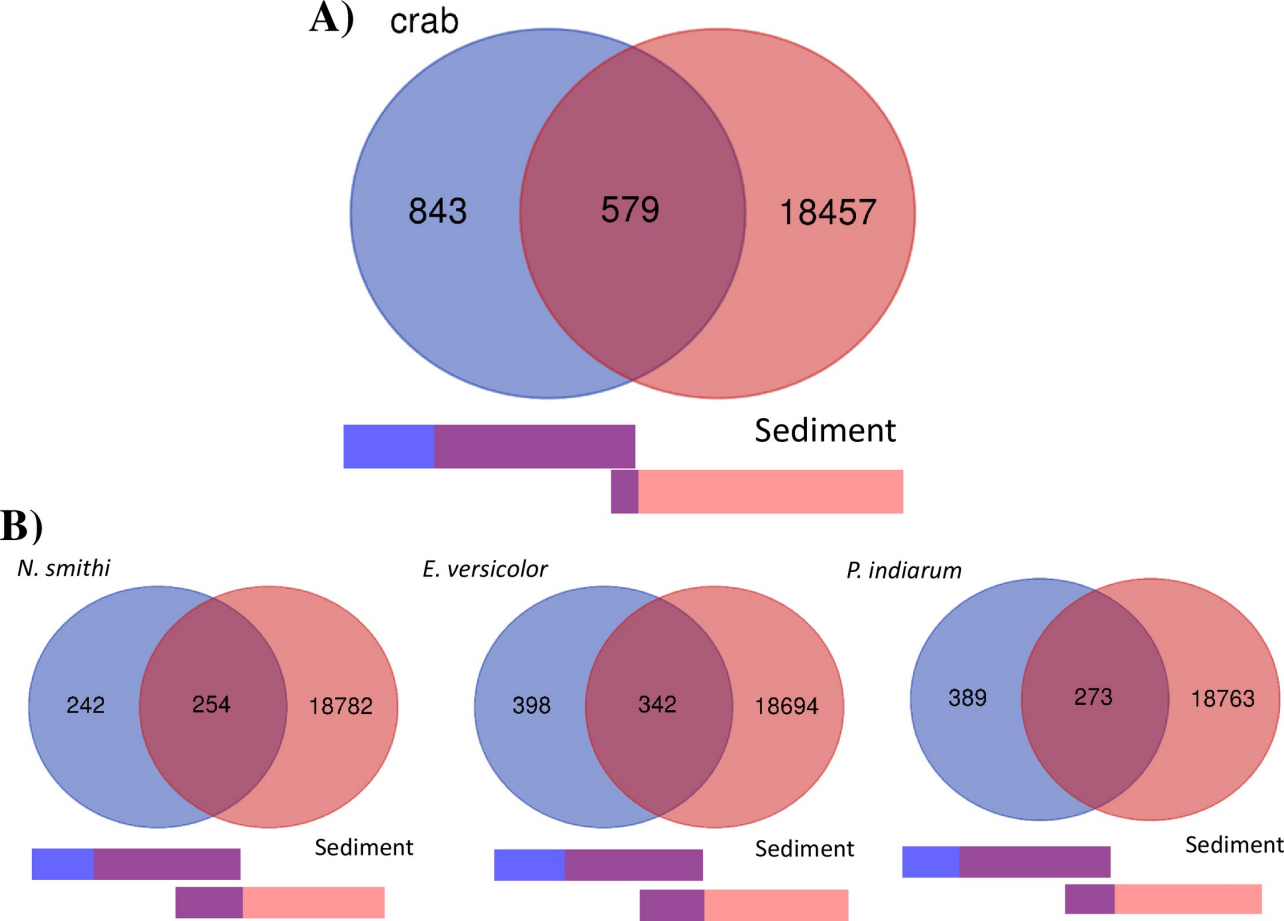
Venn diagram analysis of crab intestinal and sediment bacteria. A) Analysis of total OTUs between crab intestine and sediment samples. Bar below indicates the percentage of reads, and purple region represents the percentage of shared OTUs. B) Analysis between each of the crab species and total sediment OTUs. The shared OTUs are summarized in S7 File.

Fig 5A shows the shared and unique OTUs between the crabs and sediment. Among the total of 19879 OTUs, 579 OTUs were shared between crabs and sediment, whereas 843 and 18457 were unique to crabs and sediment, respectively. In crabs, 68.8% of reads were assigned to shared OTUs, whereas in the sediment, only 9.6% were shared OTUs. No clear differences were observed between species in the diagram (Fig 5B). It should be noted that *Demequinaceae* (OTUID 16249) was found in relatively high numbers in all crab species (S7 File; *N*. *smithi* (2.2±1.3%)*; E*. *versicolor* (2.1±2.5%); *P*. *indiarum* (1.6±1.5%)) and burrows (0.46% and 0.51% in B1 and B3, respectively). Another five OTUs for *Demequinaceae* (OTUID 662, 7688, 10605, 15748, and 469) were shared OTUs. *Oceanospirillaceae* (OTUID 2424) was another highly shared OTU that was observed solely in *N*. *smithi* (1.5±1.7%) at high content and in the burrow (0.1±0.2%).

### 3.8. Cellulase activity of the intestines of mangrove crabs and sediment

**Fig 6:**
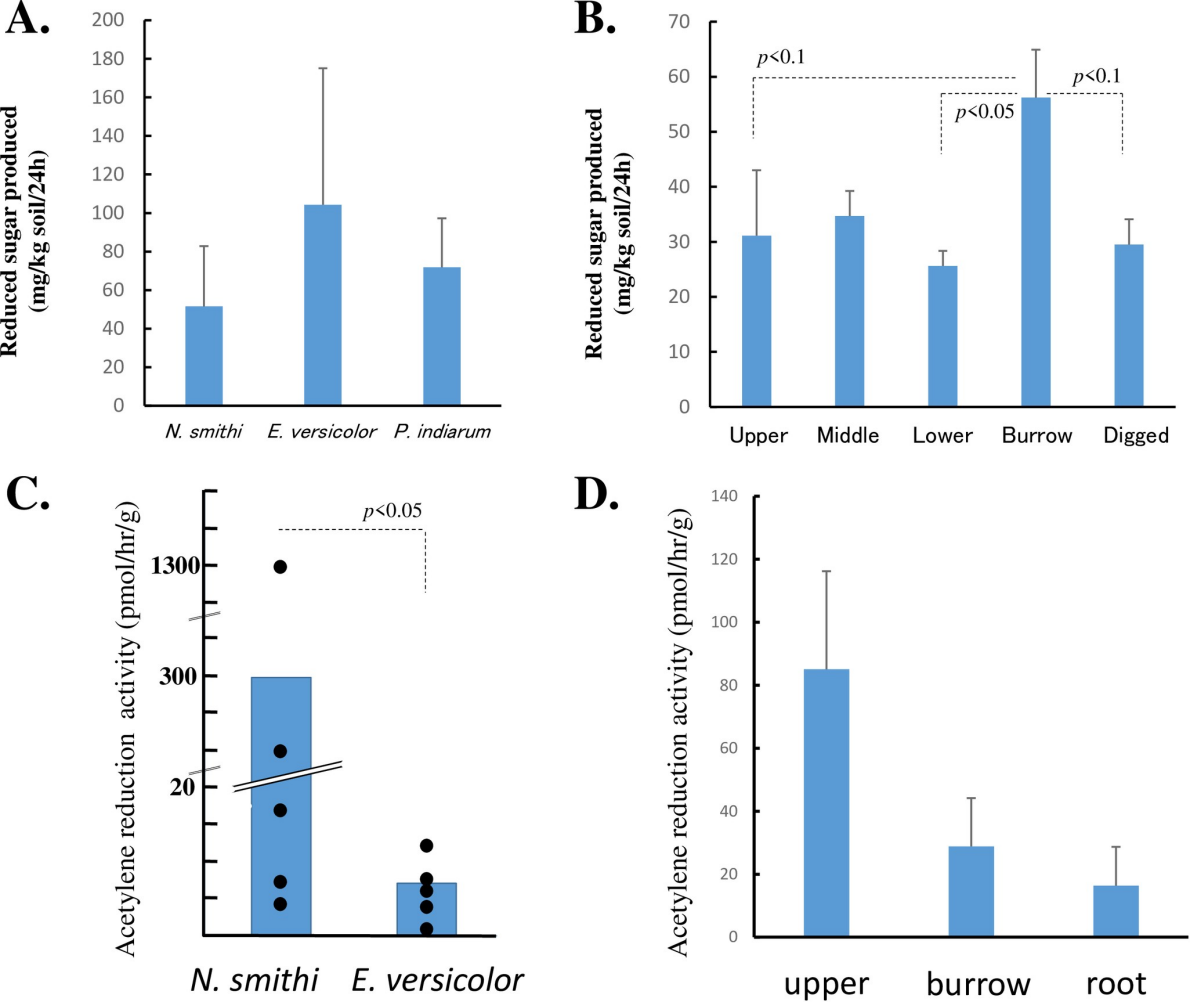
Cellulase and nitrogenase activities of the sediment and mangrove intestinal bacteria. A) Cellulase activity in crab intestines. B) Cellulase activity in sediment regions. C) Acetylene reduction activity in crab intestines. D) Acetylene reduction activity in sediment regions.

Significant activity was observed in crab intestines, regardless of the species (Fig 6A). No significant differences among species were observed in the ANOVA with a post hoc test. Fig 6B shows that apparent cellulase activity was observed in all habitat sediments where the values significantly varied depending on the five regions in the mound, based on ANOVA (*p*<0.05); the activity in the burrow was higher than that in other regions (*p*<0.05 for lower region; *p*<0.1 in dug and upper regions in Dunnett’s test), except for the middle region. Overall, the specific cellulolytic activity of intestinal bacteria was higher than that of the sediments.

### 3.9. Nitrogenase activity of the intestines of mangrove crabs and sediment

Fig 6C shows the apparent nitrogenase activity of the intestinal bacteria in *N*. *smithi* and *E*. *versicolor*, where the value was significantly higher in the former (*p*<0.05, Wilcoxon test). Notably, two *N*. *smithi* samples showed remarkably high activity (237 and 1269 pmol/h/g) compared to other individuals. Nitrogenase activity was also investigated in all habitat sediments tested, although no significant differences in activity were observed among the regions (Fig 6D). No data were available for the intestines of *P*. *indiarum* or the upper, middle, and dug sediment regions.

### 3.10. Stable isotope analysis

**Fig 7:**
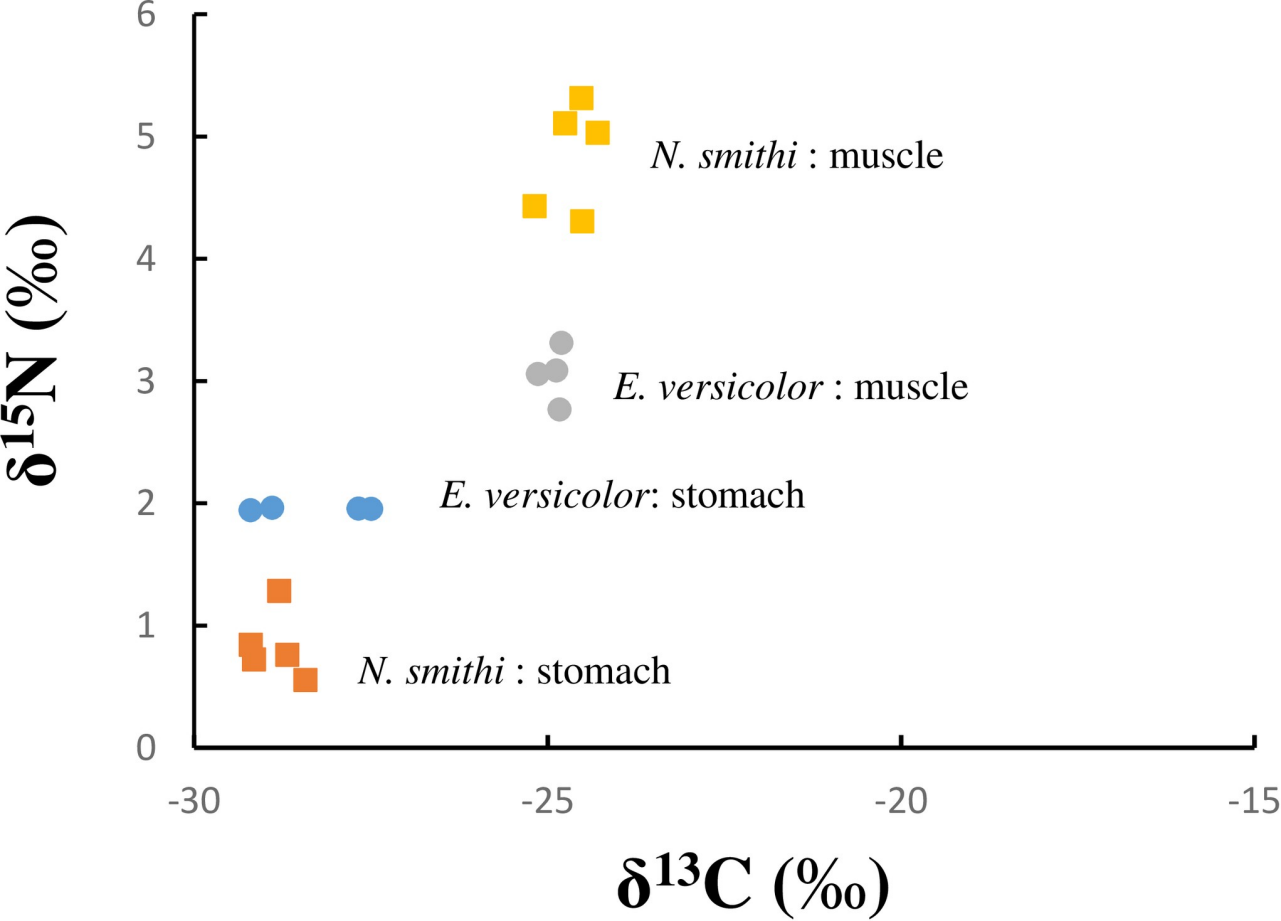
Stable isotope analysis. The X and Y axis show δ^13^C and δ^15^N values, respectively, of sesarmid crab stomach content and muscle. The symbols represent *N*. *versicolor* stomach contents (blue circle) and muscle (gray circle), and *N*. *smithi* stomach contents (orange square) and muscle (yellow square). The statistical analysis was performed using Student’s t-test.

The δ^13^C values of *N*. *versicolor* and *N*. *smithi* stomach content almost corresponded to each other (-28.32±0.74 and -28.7±0.29‰, respectively), suggesting that the food source of plant material is almost identical, while the δ^15^N values of *N*. *versicolor* (1.95±0.01‰) were significantly higher than those of *N*. *smithi* (0.86±0.24‰) (Fig 7; *p*<0.01). The difference in δ^13^C between the stomach content and the muscle was similar between crab species, with values of approximately 3.41±0.71 and 4.26±0.27‰ in *N*. *versicolor* and *N*. *smithi*, respectively, while that of δ^15^N was clearly different between species, with values of 1.10±0.20 and 4.07±0.38‰, respectively (Fig 7; *p*<0.01).

## 4. Discussion

This is the first report to identify that both sesarmid crab intestinal bacteria and habitat sediment bacteria have cellulase and nitrogenase activities. Crab intestines and habitat sediments shared a marked number of bacteria (579 OTUs), which were more enriched in the intestine (approximately 70% of reads). Here, we summarize the microbiome profiles (1.), the functions of carbon and nitrogen metabolism (2. and comparative analyses with other animal systems (3.).

### 4.1. Metagenomics profiles

#### 4.1.1. Crab intestinal microbiome

At the phylum level, Proteobacteria was dominant regardless of the species. The levels of Firmicutes of *N*. *smithi and E*. *versicolor* were significantly higher than those of *P*. *indiarum* which is mainly attributable to *Lachnospiraceae* (Fig 2A; in Wald test (alpha<0.1)). At the family level, the cellulolytic *Demequinceae* was notably higher than 3%, regardless of the species (S4 and S5 Files). Higher amounts of *Spirochaetaceae* (5.60% on average) were enriched in *N*. *smithi* in the Wald test (alpha<0.1; S4 and S5 Files). In fiddler crabs that live in mangroves, Proteobacteria and Bacteroides have been found to be enriched [28] In the present study, we found that photosynthetic *Rhodobacteraceae* was enriched in the intestine (3.7%, 4.5%, and 10.8% in *N*. *smithi*, *E*. *versicolor*, and *P*. *indiarum*, respectively). *Bacteroidetes* are highly abundant in the better-known litter feeder termites [63]. Similarly, Firmicutes are represented by common intestinal bacteria that are shared between termites and crabs [63–65]. Venn diagram and MDS analysis identified some common OTUs among crab species; however, clear species specificity, particularly for *N*. *smithi*, was found in ANOSIM test (Fig 3A and S3 File). In our field observations, the sesarmid crabs seemingly had different habitat preferences around the mounds; for example, *N*. *smithi* was found mostly in or near their burrow openings in the middle to upper part of the mound, and rarely in the flats (lower region of the mound). In contrast, *E*. *versicolor* and *P*. *indiarum* walked around the forest floor and were mostly found in the lower region of the mound. The sediment microbiome profile gradually varied depending on the region of the mound (Figs 2B and 3B and S3 File). Thus, the metagenomic profiles of the crabs are species-specific; however, the environment of the surroundings may also affect the profiles. This is supported by the fact that a particular individual of *E*. *versicolor* (EV4) collected in the sandier substrate showed a distinct intestinal microbiome (Fig 2A). The effects of the environment may occur through the microbiome of the habitat (pools of bacteria may colonize the crab intestines), as suggested in fiddler crabs [28]. In termites, however, phylogeny affects the intestinal microbial profile rather than the environment or diets [66–68]. Nevertheless, there is scope for further investigation in the future.

#### 4.1.2. Sediment microbiome

Sediment microbiome analysis revealed that Proteobacteria was the most dominant phylum, especially containing sulfur-related genera, according to previous reports (Fig 2B) [22–25]. Notably, some OTUs (*Demequinceae*) were found specifically in the burrows. In the MDS analysis, a regionally dependent cluster was observed in ANOSIM test, but the profile was not perfectly conserved between years (2013–2014) (Fig 3B). The OTUs were highly shared between the middle and lower regions, followed by the middle region and burrow, which were physically close (S3 File). This distribution pattern is presumably regulated by key physicochemical factors in the mound. For example, the upper region is rarely immersed by tides, except for the timing of high tides, while the flat region was regularly submerged twice per day. This suggests that the upper region is more arid and provides breathable and salty conditions for bacteria [5, 28, 69]. In addition, the upper region is more easily affected by sunlight because it has fewer obstacles apart from mangrove trees and leaves. Thus, it is reasonable that the upper region showed specific profiles that were statistically characterized by *Hyphomicrobiaceae*, *Mariprofundaceae*, and *Rhodobacteraceae* in the Wald test (alpha<0.1). These three families were aerobic. Zhu et al. (2018) reported that the upper zone was occupied by higher proportions of heterotrophic bacteria, such as *Desulfobacterales*, *Anaerolineae*, and *Acidobacteria*, while the proportion of *Rhodobacterales* and *Xenococcaceae* was greatly increased in the lower zone [69]. Thus, a distinct microbiome pattern may ubiquitously occur between the upper region and flat zones in the intertidal region, but the profile may vary depending on the environment and field [69]. In the upper region, sporadically high values were observed in some families (*Ignavibacteriaceae*, *Ectothiorhodospiraceae*, *Acetobacteraceae*, *Acidobacteriaceae*, and *Conexibacteraceae*).

### 4.2. Functional analysis of microbiomes in the intestine and sediment

#### 4.2.1. Metabolism of carbon

It is generally accepted that sediment microbes decompose cellulose in litter before transportation of organic matter by tides [5, 11, 14, 70]. In a previous report, we identified β-1,4-endoglucanse, β-glucosidase, and total cellulase activity in *E*. *versicolor*, *P*. *indiarum*, and *Episesarma palawanense* [39]. Bui and Lee (2015) reported the same enzymatic activities in *Parasesarma erythodactyla* and determined the complete/partial cDNA sequences of putative endo- β -1,4-glucanase in nine mangrove crabs [38]. These data suggest that mangrove crabs can endogenously digest cellulose, regardless of the species. These results are supported by the present results (Fig 6A and 6B). The litter is first shredded by the mouth/stomach teeth, which can promote the easy accessibility of the enzyme, and the cellulose involved is degraded in the stomach by endo- β-1,4-glucanase and β-glucosidase in the digestive juice. Partially degraded cellulose is then degraded by endo-β-1,4-glucanase from intestinal bacteria. After being released as feces in the sediment, cellulose was further degraded by cellulase from the sediment bacteria.

Kristensen and Pilgaard (2001) reported the degrading effect of deposited fecal pellets from *N*. *versicolor* fed with green *R*. *apiculata* leaves on the heterogeneity of anaerobic microbial activity in sediment [17]. The solid fecal material supported a 55-fold faster microbial decay than the solid leaf material. Similar studies have also been conducted, showing that sesarmid crabs reduce mangrove leaf litter to fecal fragments, resulting in faster microbial colonization, which enhances the breakdown of mangrove detritus, nutrient recycling, and retention in mangrove sediment [16, 71]. Our results support these data from the viewpoint of cellulose degradation in litter. During the degradation in the sediment, the more degraded the cellulose in the feces, the higher the water solubility because the molecular weight and solubility of cellulose are highly correlated [33]. Considering that dissolved organic carbon (DOC) derived from litter plays a key role in the food web as a main source after being transported offshore, mangrove crabs and sediments have an immense impact on the outwelling patterns of a mangrove ecosystem [14, 72–75].

Stable isotope data in the present study showed that both the δ^13^C values of the stomach contents of *N*. *smithi* and *N*. *versicolor* were approximately -29‰ (Fig 7). In Kristensen et al. (2017), various environments worldwide were tested for stable isotope analysis: δ^15^N of the mangrove litter ranged from 1.6 to 6.6‰, δ^13^C ranged from -30.8 to -25.0‰, whereas in MPB, the values were 2.3‰ and ranged from -20 to -19‰ [11]. Applying these data to the present study, the main food and carbon sources in the stomach contents of these crabs were litter. The difference in δ^13^C between stomach content and muscle was approximately 3.41±0.71 and 4.26±0.27‰ in *N*. *smithi* and *E*. *versicolor*, which was much higher than that of universally used discrimination values (0.4‰) per trophic level, but similar to previous studies (about 5‰) [11, 41, 45, 76]. This indicated that carbon metabolism, including assimilation, was similar to that reported previously. The link between this high discrimination value for carbon in the digestive manner of cellulose in the digestive juice and intestinal bacteria remains unclear. Kawaida et al. (2019) reported a close relationship between the assimilation efficiency of plant materials as a carbon source and the level of cellulase activity in the hepatopancreas using six crab species in the mangrove [45]. If cellulose is degraded by digestive enzymes and the intestine before assimilation, further examination is necessary.

*Demequinaceae*, found in crabs and burrows, is the most potent candidate for cellulose-degrading bacteria, and is closely related to *Cellulomonadaceae*, a family of cellulolytic bacteria (S4, S5 and S7 Files). This family (*Demequinaceae*) was highly and uniformly distributed in the three mangrove crabs (over 3% regardless of the species) and was enriched in the mound burrow (1.6% and 1.3% in B1 and B3, respectively). The *Demequinaceae* family of the order *Actinomycetales* was first proposed by Yi et al. (2007), who described a single recognized species, *Demequina aestuarii* [77, 78]. To date, this genus contains eight species; however, little information is available on the cellulose degradation activity of this genus, except for *D*. *aestuarii*. Nevertheless, from the genomes of six *Demequina* species, proteins belonging to glycoside hydrolase (GH) families were annotated (not shown). In the present study, *Demequinaceae* was also detected in the sediment, although the percentage (average 0.15%) was much lower than that in the intestinal microbiome (average 3.97%) (S4, S5 and S7 Files). However, the percentages in the burrows were relatively high (1.6% and 1.3% in B1 and B3). The crabs normally live in the burrow and regularly defecate. The lack of *Demequinaceae* detected in B2 might be ascribed to the low coverage of this sample or the unequal distribution of feces in the wall of the burrow. According to the phenotypes in the metagenome analysis, the cellulose-degrading activity in the intestine can be attributed partly to the *Bacteroides* or *Clostridium* genera in METAGENassist. In the phenotype prediction (Fig 4), a very low percentage of OTUs was enriched in “cellulose degrader” in the sediment, but this was not confirmed by the cellulase assay (Figs 4B and 6). It should be noted that these *Demequinaceae* reads were not assigned to cellulose degraders in this system because *Demequinaceae* was a newly identified family (Fig 4). Although cellulose degradation by fungi in mangrove sediments has been described [35], fungi were not detected through 16S metagenome analysis in this study. However, to the best of our knowledge, little is established about their symbiosis with crabs.

Thus, the present study shows the cooperative occurrence of cellulose degradation in both the intestine and sediment microbiome as well as endogenous cellulase in the digestive juice. The public carbohydrate-active enzyme (CAZyme) database includes 156 different families of GHs based on their structure and function (Lombard et al., 2014). In ten GH families (GH1, 3, 5, 6, 7, 8, 9, 12, 45, and 48), cellulolytic activity was found (15 families for hemicellulose). Thus, the robust cellulose molecule is degraded by a vast array of specific activities of highly diverse enzymes [79]. Moreover, sequentially associated reactions by a variety of GHs in endogenous enzymes in the intestinal and sediment microbiomes may affect the rate of degradation (solubility) of cellulose before transporting organic compounds into the ocean. Bui and Lee (2014) reported that cellulolytic enzymes in mangrove crabs were classified as GH9 [38] using the genome of *Demequinaceae* available in the database, GH3 was found. The classification of GHs in the present study samples remains unclear, but several GHs may be involved sequentially in cellulose degradation in the mangrove ecosystem.

#### 4.2.2. Metabolism of nitrogen

To date, N_2_-fixation in mangrove ecosystems has been detected mainly in sediments [5, 46, 49–53]. Similarly, we also detected nitrogenase activity in the upper, burrow, and root regions of the mound (Fig 6D). Based on the data in the phenotype prediction analysis of the intestinal microbiome of the crabs, we also detected apparent nitrogenase activity in the intestinal bacteria of *E*. *versicolor and N*. *smithi* (Fig 6C). In animals, nitrogenase activity has been reported in the symbiotic microbes in termites, wood-boring beetles, shipworms, sponges, corals, and fiddler crabs, which allow them to survive in nitrogen-poor environments [26, 80–83]. Generally, nitrogen-containing compounds are partly released as feces, while other compounds are incorporated into the body of the host animal [65]. According to Tayasu et al. (1994), at least 30%–60% of the nitrogen content of the wood-feeding termite *Neotermes koshunensis* (Kalotermitidae, Isoptera) is derived from the atmosphere via nitrogen fixation [84]. Moreover, Thompson (2013) estimated that the wood-feeding sawfly *S*. *noctilio* F. derives >90% of its larval N budget from N_2_ fixation [85].

However, it is still premature to apply these cases directly to sesarmid crabs because an earlier study ruled out the assimilation of symbiotic nitrogen-fixing bacteria in the intestine, based on data obtained by a CHN analyzer after a breeding experiment of *N*. *versicolor* [86]. The prevailing opinion so far, based on analysis including stable isotope analysis, is that most leaf-eating sesarmid crabs compensate for their N supply partly by occasional consumption of animal tissues, such as carcasses of fish, crustaceans, and mollusks [87, 88], or through predation/cannibalism, and partly by sediment-associated bacteria, MPBs, fungi, and meiofauna [89–91].

In the present study, the stable isotope analysis did not support the prediction that crab muscle shows lower δ^15^N than gut contents (food) of crabs, possibly due to nitrogen fixation in intestinal microbes and assimilation of microbes (i.e., δ^15^N value was close to zero) (Fig 7). In the biofilm on the carapace of the fiddler crabs, δ^15^N values were significantly lower than in primary producers as potential sources of detritus (phytoplankton, MPBs, litter, etc.), although the values from muscle and other tissues were approximately 8% [26], which is similar to or higher than the values reported in the present study. The present findings indicate the contribution of nitrogen fixation from the intestinal bacteria of sesarmid crabs; false positives from the assay are rare because the reaction requires high activation energy. Further investigation is needed to analyze the contribution of the intestinal microbiome to the body.

In *N*. *versicolor* and *N*. *smithi*, a clear difference in Δ^15^N values between the stomach content and muscle was observed, with values of 1.10±0.20‰ and 4.07±0.38‰, respectively (Fig 7; *p*<0.01 in t-test). This may reflect the digestive systems of both the crab species. Using the isotope mixing model, Kristensen et al. (2017) reported that Δ^15^N in leaf-eating crabs may vary up to 3‰ without serious problems [11]. The δ^15^N values of the stomach contents of *N*. *versicolor* were significantly higher than those of *N*. *smithi* (Fig 7; *p*<0.01 in t-test). This may be because *N*. *smithi* are more herbivorous, but the aging of litter and the crab preference for leaf color should be considered. The litter changes in color from green to yellow-brown, and there have been several reports about crab preference for this color/aging [43, 88, 92]. *N*. *versicolor* reportedly prefers brown litter to other colors, whereas *N*. *smithi* shows little preference for leaf color. In earlier studies, δ^13^N values generally decreased with aging, suggesting that we cannot easily compare these values between species to determine their food composition [88, 92].

In the phenotype prediction for nitrogen fixation, the enriched OTUs were mainly derived from the families *Spirochaetaceae*, *Shewanellaceae*, *Vibrionaceae*, and *Rhodobacteraceae* (Fig 4). The presence of *Spirochaetaceae* is noteworthy. In the termite gut, nitrogen-fixing and ammonia-oxidizing bacteria play a crucial role in providing termite bodies with nitrogen from the low nitrogen content of the wood diet [93]. It is widely accepted that *Spirochetes*, the most conspicuous bacterial group in lower termite guts, is capable of diverse metabolic processes, including nitrogen fixation, acetogenesis, and degradation of lignin phenolics (Lilburn et al., 2001; Warnecke et al., 2007; Yamada et al., 2007; Hongoh et al., 2008; Lucey and Leadbetter, 2014) [94–98]. It is important to note that a significantly higher percentage of *Spirochaetaceae* was observed in *N*. *smithi* (5.60%), than in *E*. *versicolor* (<1.1%) and *P*. *indiarum* (0.3%) (Wald test; alpha<0.1; S4 and S5 Files). In addition, the nitrogenase activity in *N*. *smithi* was higher than that in *E*. *versicolor*, with remarkably high activity in the two individual samples (*p*<0.05, Wilcoxon test; Fig 6C). It should also be noted that, based on previous reports and our visual observations, *N*. *smithi* is herbivorous, while the other two species are omnivorous [43, 92]. The function of *Spirochaetaceae*, especially *N*. *smithi*, should be further investigated in the future.

Nitrogen in the air can be fixed in both the sediment and the crab intestine. It should be noted that the activity of nitrogenase is generally quite fragile against oxygen and is easily inactivated within a few days [99, 100]. The intestinal condition is anaerobic; however, once it is released into the sediment, it is exposed to oxygen [100]. It has been generally suggested that crabs eat their feces mixed with soils, resulting in repeated processing in this step [18, 70, 101]. After the crabs eat the mixture containing the bacteria, the intestine can provide anaerobic conditions to the bacteria again. If this is repeated, it might be possible that the viability of the bacteria is maintained to fix nitrogen. The reason for the two outliers in nitrogenase activity in *N*. *smithi* remains unknown (Fig 6C), but in plants, the induction of nitrogenase at the transcriptional level under nitrogen-limited conditions has been reported [102]. Although the details remain unknown, nitrogenase activity might be suppressed under normal conditions but stimulated by unknown factors.

In the phenotype analysis of the sediment, nitrogen cycles such as ammonia oxidizer, nitrate reduction, and nitrogen fixation were enriched regardless of the mound regions (Fig 4B). Similar results were obtained from PICRUSTs (S6 File). These findings may be mainly attributable to *Desulfobulbaceae*, *Rhodobacteraceae*, *Hyphomicrobiaceae*, etc., in the analysis using METAGENassist. This partly follows Andreote et al. (2012), who found that the microbial core involved in methane, nitrogen, and sulfur metabolism consists mainly of *Rhodobacteraceae*, *Desulfobacteraceae*, *Burkholderiaceae*, and *Planctomycetaceae* [25]. The samples used to determine nitrogenase activity and perform metagenome analysis were obtained during different years and months (November 2017 and Dec 2013/2014, respectively); thus, environmental factors could differ and influence the results. However, the study area is characterized by a long rainy season from May to December, and the average temperature in November and December was almost constant (low-high: 21–32°C) [103]. Thus, temperature and precipitation conditions are unlikely to differ significantly between the samples. Spring-neap tidal cycle might play a role because the samples were taken at different periods of the cycle, where the frequency of inundation/exposure differs. Further studies considering temporal changes in the metagenome and enzymatic analyses are needed.

#### 4.2.3. Metabolism of sulfur

Under anaerobic conditions in mangrove sediment, it is widely accepted that oxygenic sulfur-oxidizing bacteria and strict anaerobic sulfate-reducing bacteria (SRBs) are very important in the processing [24] where SRBs degrade organic compounds, including cellulose, by using sulfate as a terminal electron acceptor [104–106]. In marine sediments from temperate climates, SRBs degrade 53% of organic matter, and this value varies between 70% and 90% in salt marsh plateaus [104]. There are many reports about the dominance of sulfur-reducing bacteria in mangrove sediments, which are classified as δ-Proteobacteria and ε-Proteobacteria [24, 104], although little is known about the function of intestinal bacteria in sulfur metabolism. In the present study, sulfur-related *Desulfobulbaceae* (δ-Proteobacteria; 16.2±5.0%), *Desulfobulbaceae* (δ-Proteobacteria; 10.2±5.4%), *Desulfarculaceae* (δ-Proteobacteria; 3.9±1.5%), and *Desulfuromonadaceae* (2.2±1.5%) were highly enriched in the sediment, whereas they were clearly less enriched in crab intestines (0.02±0.04%, 1.4±1.1%, 0.02±0.07%, and 0.10±0.2%, respectively) (S5 File). The completely inconsistent levels of *Desulfobacteraceae*, *Desulfobulbaceae*, *Desulfarculaceae*, and *Desulfuromonadaceae* may suggest mutual complementation of sulfur metabolism between the intestine and sediment, but this remains unclear.

#### 4.2.4. Significance of the burrow

From our observations during the field sampling, the burrows sampled in this study were considered *N*. *smithi* burrows. They showed higher cellulase activity than other regions in the Dunnett’s test (Fig 6B), and a significant number of OTUs were enriched in phenotype prediction (Fig 4B). *N*. *simithi* mainly inhabits the mid to upper part of the lobster mound, whereas burrows of *E*. *versicolor* and *P*. *indiarum* are usually on the flat or root area of the mangrove (not collected in this study). In addition, the body and corresponding burrow entrance size of *N*. *smithi* were larger than those of other species. As per the Venn diagram analysis of the burrow and three crab intestines (S8 File), a total of 349 burrow OTUs were shared between the crabs, including *Demequinceae*. Eighty OTUs were specifically shared with *N*. *smithi*, which included *Spirochaetaceae and Desulfobulbaceae*. Indeed, crabs mainly feed on the litter in the burrow, but it is still not wise to discuss the species-specific metabolic function of the burrow because we did not have the data of the burrow of *E*. *versicolor* and *P*. *indiarum*.

In the experimental design, the burrow in this study was expected to be most bioturbated, while lower (open space) regions were expected to be less affected by animals. However, 2045, 3071, 2524, and 2417 OTUs from burrows were shared with the upper, middle, lower mound, and dug regions, respectively, in the Venn diagram analysis (S3 File). Considering the OTU sharing with crab intestine (S7 and S8 Files), the burrow may be the most bioturbated, but the effects were not restricted to this region, possibly because of the mixing of the sediment by tidal effects or some other factors.

### 4.3. Comparative analysis of the associated metabolic roles

In this study, we identified the relationship between the sesarmid crab intestinal and burrow microbiomes and investigated their associated roles in carbon and nitrogen metabolism. A similar effect was reported in fiddler crabs in mangroves and other animals. Cueller-Gempeler and Leibold (2018) investigated the relationships between distinct regions of habitat sediment (from burrow, subsurface, and surface) and crab-associated bacteria (gut and carapace) using metagenomic techniques [28]. All carapace OTUs were identical to the burrow and 10% of the gut, suggesting that the carapace and gut bacterial communities originated from the burrow sediment, although the latter was partly from the surface sediment. Their report mainly focused on the carapace, but emphasized that crab activity can interactively modulate the diversity and composition of burrow sediment bacterial communities. Booth et al. (2019) reported that burrowing by fiddler crabs can induce bioturbation effects on the microbiome of the mangrove environment, irrespective of the sampling site [27]. They insisted that the burrow could regulate the oxygen concentration and redox potential, although they did not investigate the gut microbiome of crabs. In addition, Zilius (2020) reported that the association of fiddler crabs in the mangrove and their carapace functions as hot spots of microbial N metabolism in a microcosm experiment [26]. According to their report, the δ^15^N value of the carapace was clearly lower than that of the primary producer and that of other crabs, which supports the idea of nitrogen fixation in the air. Thus, the functional association between fiddler crab symbiotic bacteria and that of sediment is similar to that between the sesarmid crab and the sediment in many aspects.

However, there are clear differences between fiddler crab and sesarmid crab ecology (habitat preference, size of burrow morphologies, food preferences, etc.). The most marked difference was foraging behavior in the sesarmid crabs. While sesarmid crabs handle and feed on massive litter, fiddler crabs are surface feeders that rely mainly on MPBs and bacteria and do not feed on litter, suggesting that the diets and digestive systems, especially in relation to carbon, are distinct.

Apart from mangrove crabs, there are reports that show associations with the metabolic systems of invertebrate microbes. In termites, Brauman (2000) pointed out that the mounds of soil-feeding *Cubitermes niokolensis* were built from a combination of feces and soil, which contained significantly higher amounts of organic (C and N) and inorganic nutrients (P, Ca, and NH_4_) than those in adjacent soils. Fungus-growing termites mix plant matter with fungal symbionts in the gut and deposit the blend where the organic matter is decomposed [107]. After the fungus produces nodules, the termites feed on it. Otani et al. (2016) investigated the microbiome of the fungus comb and gut of termites and found that large proportions of gut bacterial communities were shared with fungal combs [29]. In contrast, fungal comb communities contain relatively small proportions of bacterial families that are present in termite guts, probably because fungal combs can be affected by their surroundings. In addition to Insecta, earthworms are widely accepted as key players in the ecosystem that affect soil moisture, gas diffusivity, and nutrient dynamics [108]. Through these modifications, earthworms can alter the structure and function of the soil microbiome [32].

Thus, there have been many reports similar to those of crabs and burrow bacterial profiles, regardless of the species and ecosystems, showing the interaction between symbiotic and surrounding microorganisms, including fungi. However, the details remain unknown, especially the microbial interactions (OTU-OTU interactions) between these factors. Metatranscriptomic analysis of the microbiome can provide further insight into the cooperative role of crabs and sediment microbes in the carbon and nitrogen cycles of the mangrove ecosystems. Here, we show a “snapshot” of the microbiome profiles, but a longer-term study is essential to understand the dynamic profiles and functions of the mangrove ecosystem.

## 5. Conclusions

**Fig 8:**
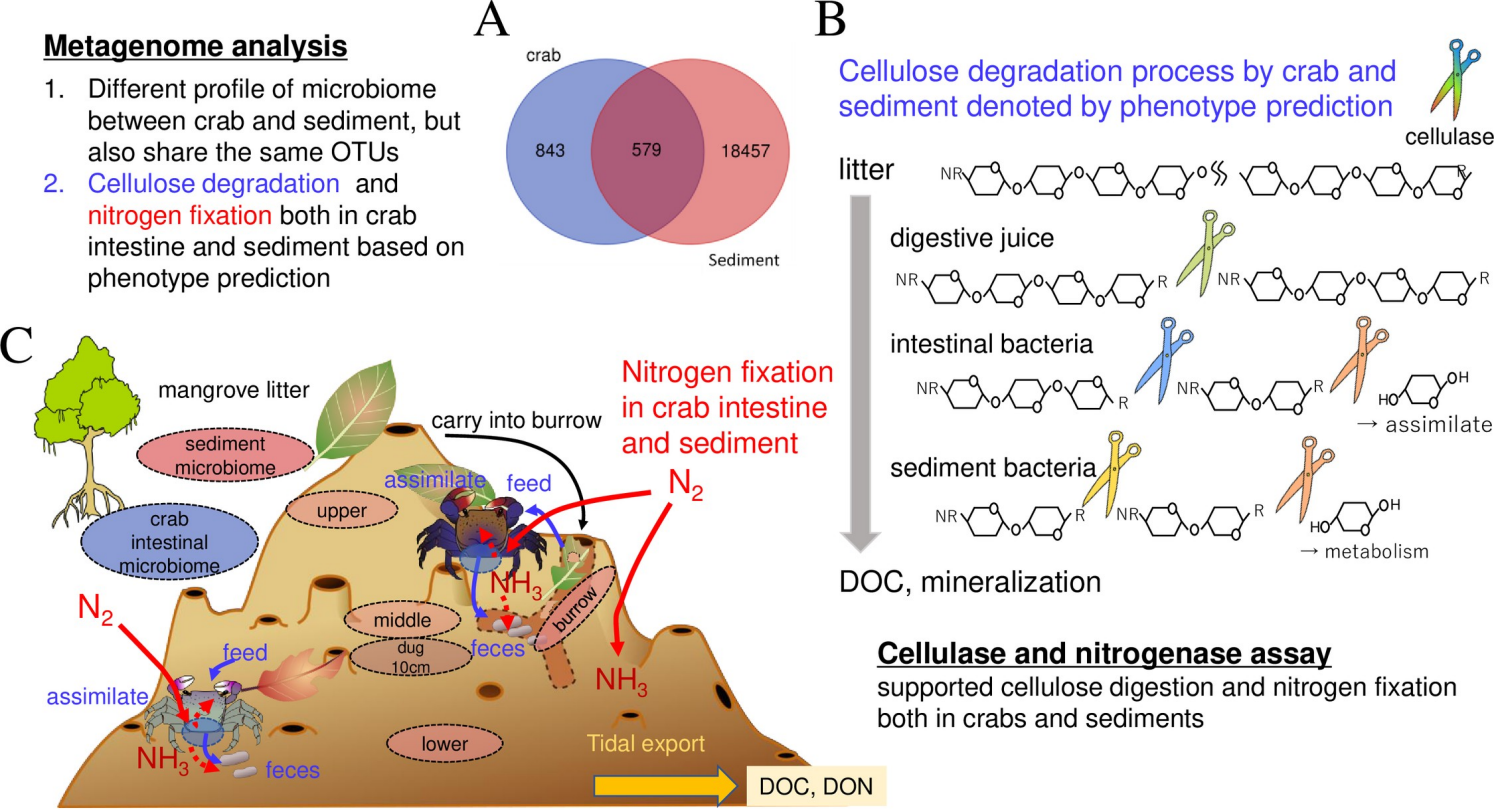
Summary of the present study. The microbiomes of the intestines and habitat sediments of leaf-eating mangrove crabs were investigated. More than 500 operational taxonomic units (OTUs) were shared between crab intestine and habitat sediment microbiomes (A). The data also predicted the occurrence of cellulose-degrading and nitrogenase activities in the intestines and sediments, which was validated by enzymatic assays. These data support the suggestion that cellulose in mangrove litter is sequentially degraded by the digestive juice and intestinal and sediment bacteria partly assimilated by the crabs (B). N_2_ in the air can be fixed in the intestines and sediments, and can be assimilated by the crabs. Degraded cellulose and fixed nitrogen in the sediment function as dissolved organic carbon (DOC) and dissolved organic nitrogen (DON) after tidal transportation into ocean water (C).

Fig 8 summarizes the highlights of this study. Here, we investigated the microbiomes of intestines from sesarmid crabs (three species) and their habitat sediments (five regions), including crab burrows, to elucidate their cooperative roles in carbon and nitrogen metabolism. The intestinal microbiomes showed some species specificity, but some OTUs were shared between species, while those in the habitat sediments showed a gradual distribution depending on physicochemical factors. Of the crab intestinal microbiome (1422 OTUs identified), almost 70% of the reads (579 OTUs) were assigned to the sediment, regardless of the species. Based on the phenotype prediction, we detected significant activities of cellulase and nitrogenase in both the crab intestine and sediment. These findings indicate the cooperative roles of foraging crab-sediment bacteria in carbon and nitrogen cycling. It is widely accepted that sediment and intestinal microbiomes can respond to environmental changes, but the details, including microbial interactions (OTU-OTU interactions) and metatranscriptomic analysis, remain unclear. In the present study, we only have a “snapshot” of the sediment and intestinal microbiomes, but further study is essential to understand their dynamic cooperative roles in turbulence over a long time scale.

## Supporting information

S1 File The OTUs obtained in this study. (XLSX)

S2 File Rarefaction curve for A) crab intestine and B) sediment.
(PPTX)

S3 File Venn diagram analysis of five sediment regions and three crab species. Each circle represents the union of OTUs for each sediment region and crab species. A) Crab intestine. B) Sediment region. The matrix below indicates the total number of OTUs shared between the regions.
(PPTX)

S4 File Family distribution and relative abundance tags were classified using a threshold of 97%. The bars represent the relative abundance of each sample. A) Crab intestine. B) Sediment region.
(PPTX)

S5 File Data for phylum and family distribution in crab intestine and sediment region. (XLSX)

S6 File Phenotype analysis using Picrust. (PPTX)

S7 File List of shared OTUs between the crab intestinal and sediment bacteria shown in Fig 5. (XLSX)

S8 File Venn diagram analysis of the burrow and three crab species. (PPTX)
