# Correction: Mangrove crab intestine and habitat sediment microbiomes cooperatively work on carbon and nitrogen cycling

**DOI:** 10.1371/journal.pone.0315952

**Published:** 2024-12-12

**Authors:** Prasert Tongnunui, Yuki Kuriya, Masahiro Murata, Hideki Sawada, Michihiro Araki, Mika Nomura, Katsuji Morioka, Tomoaki Ichie, Kou Ikejima, Kohsuke Adachi

The first author’s name is spelled incorrectly. The correct name is: Prasert Tongnunui. The correct citation is: Tongnunui P, Kuriya Y, Murata M, Sawada H, Araki M, Nomura M, et al. (2021) Mangrove crab intestine and habitat sediment microbiomes cooperatively work on carbon and nitrogen cycling. PLOS ONE 16(12): e0261654. https://doi.org/10.1371/journal.pone.0261654.
