# Supplementary material for: Mangrove crab intestine and habitat sediment microbiomes cooperatively work on carbon and nitrogen cycling
Source: PLoS One. 2021 Dec 31;16(12):e0261654. doi: 10.1371/journal.pone.0261654 (PMC8719709; doi:10.1371/journal.pone.0261654)
Supplement: S2 File — Rarefaction curve for A) crab intestine and B) sediment. (PPTX) [file pone.0261654.s002.pptx]

## Slide 1
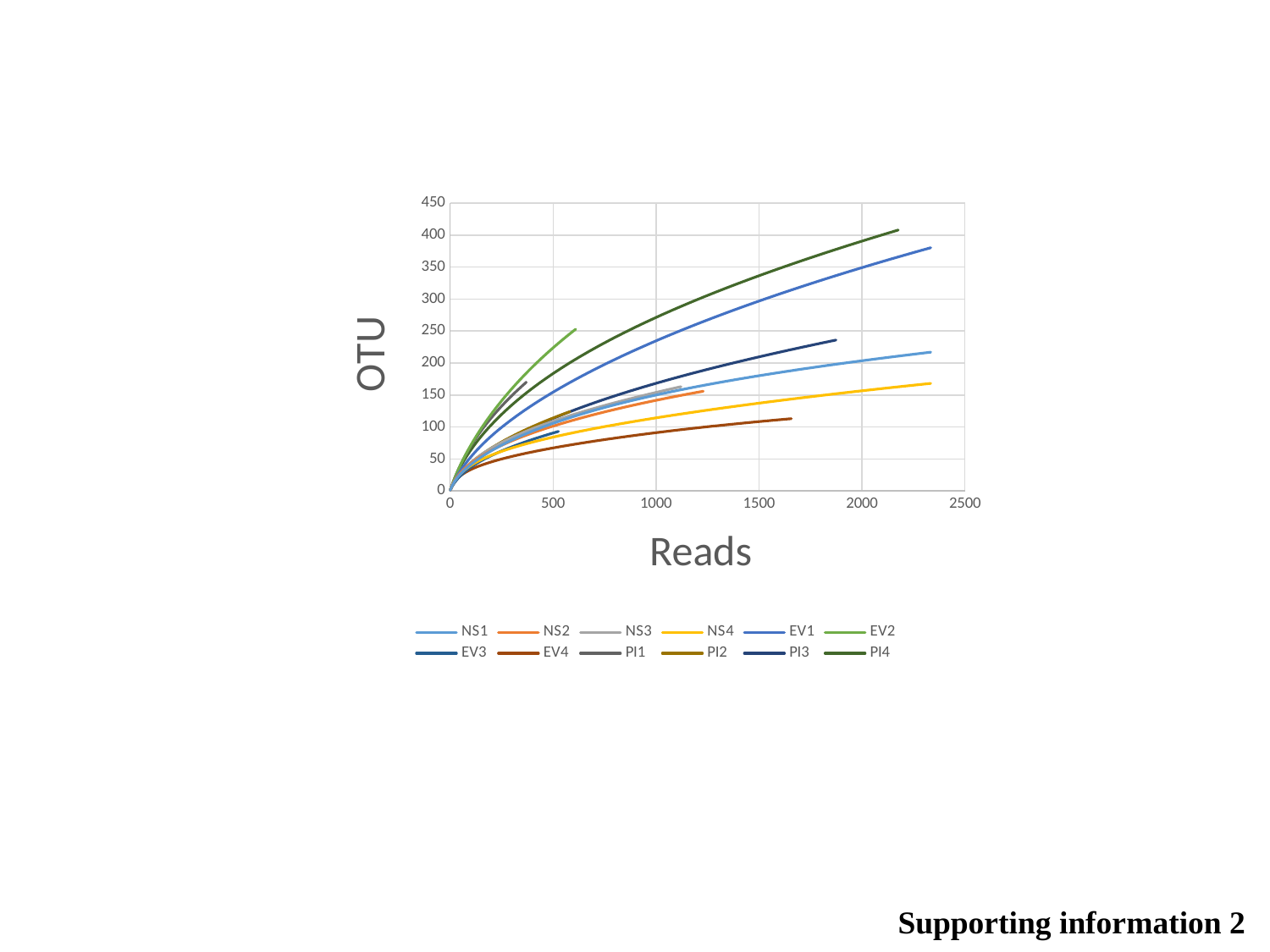

### Chart
| Category | NS1 | NS2 | NS3 | NS4 | EV1 | EV2 | EV3 | EV4 | PI1 | PI2 | PI3 | PI4 |
|---|---|---|---|---|---|---|---|---|---|---|---|---|Supporting information 2

## Slide 2
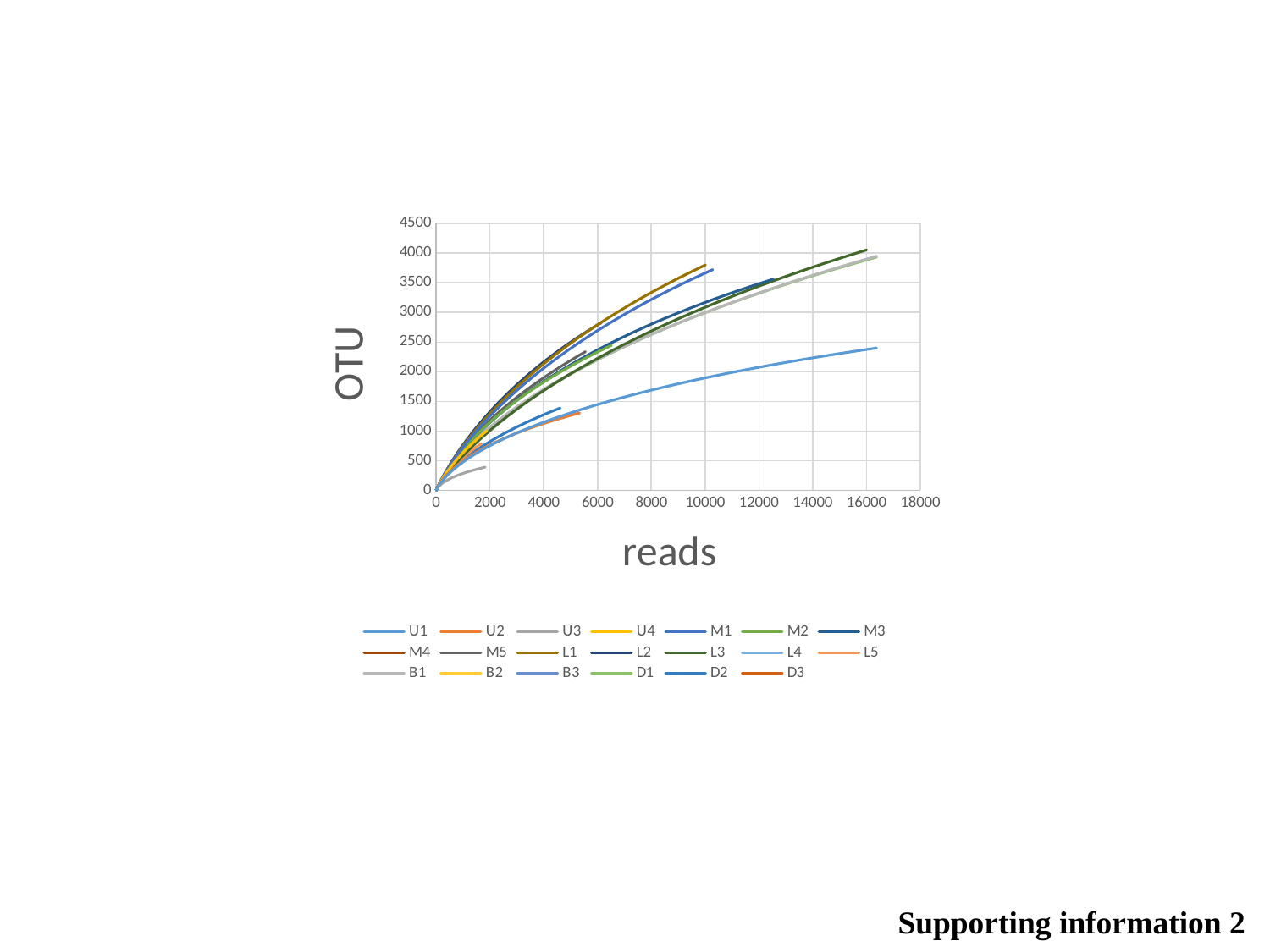

### Chart
| Category | U1 | U2 | U3 | U4 | M1 | M2 | M3 | M4 | M5 | L1 | L2 | L3 | L4 | L5 | B1 | B2 | B3 | D1 | D2 | D3 |
|---|---|---|---|---|---|---|---|---|---|---|---|---|---|---|---|---|---|---|---|---|Supporting information 2
