# Supplementary figures and images for: Mangrove crab intestine and habitat sediment microbiomes cooperatively work on carbon and nitrogen cycling
Source: PLoS One. 2021 Dec 31;16(12):e0261654. doi: 10.1371/journal.pone.0261654 (PMC8719709; doi:10.1371/journal.pone.0261654)

## Slide 1
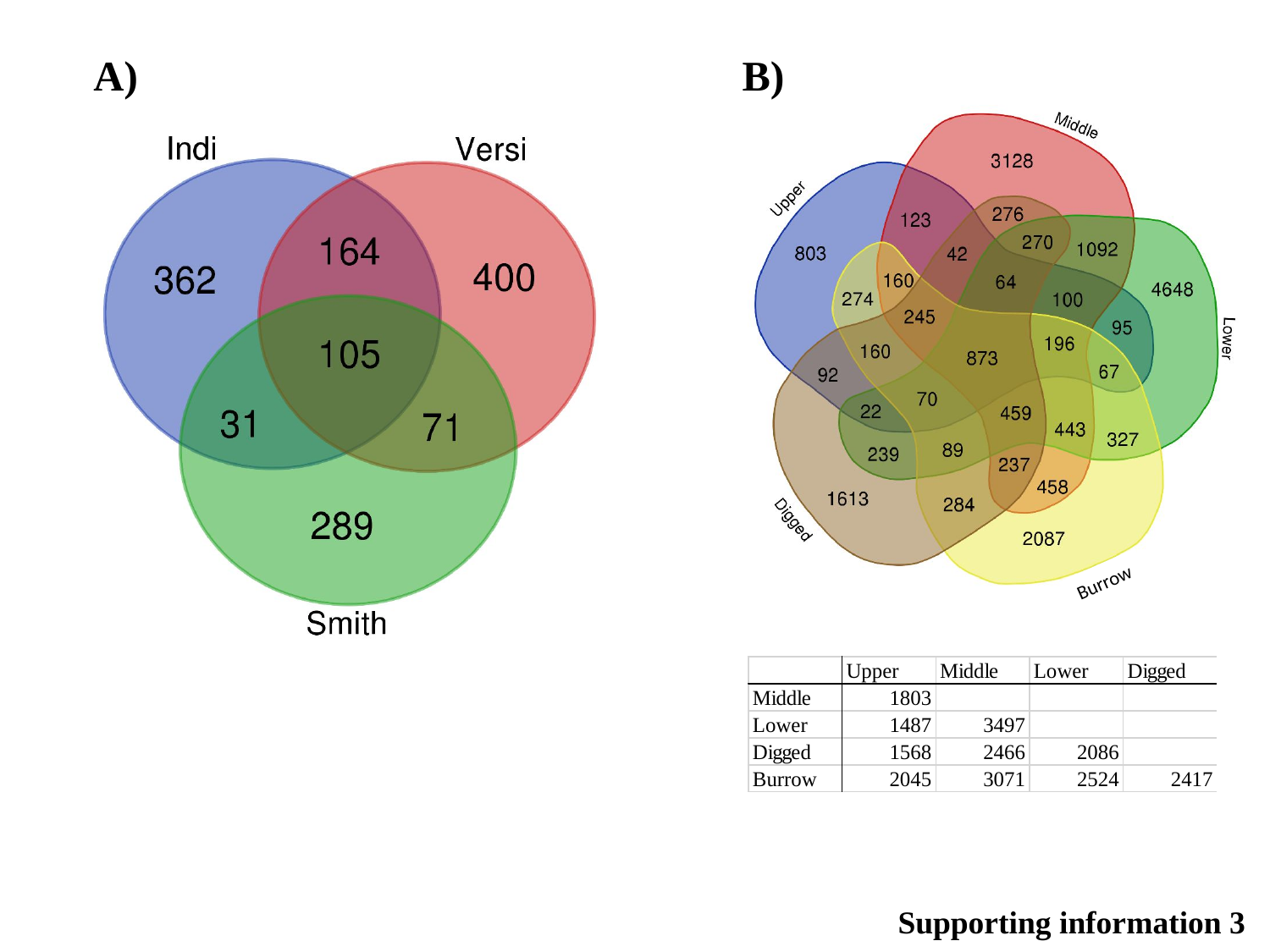

A)
B)
Burrow
Supporting information 3

Supplement: S3 File — Each circle represents the union of OTUs for each sediment region and crab species. A) Crab intestine. B) Sediment region. The matrix below indicates the total number of OTUs shared between the regions. (PPTX) [file pone.0261654.s003.pptx]

## Slide 1
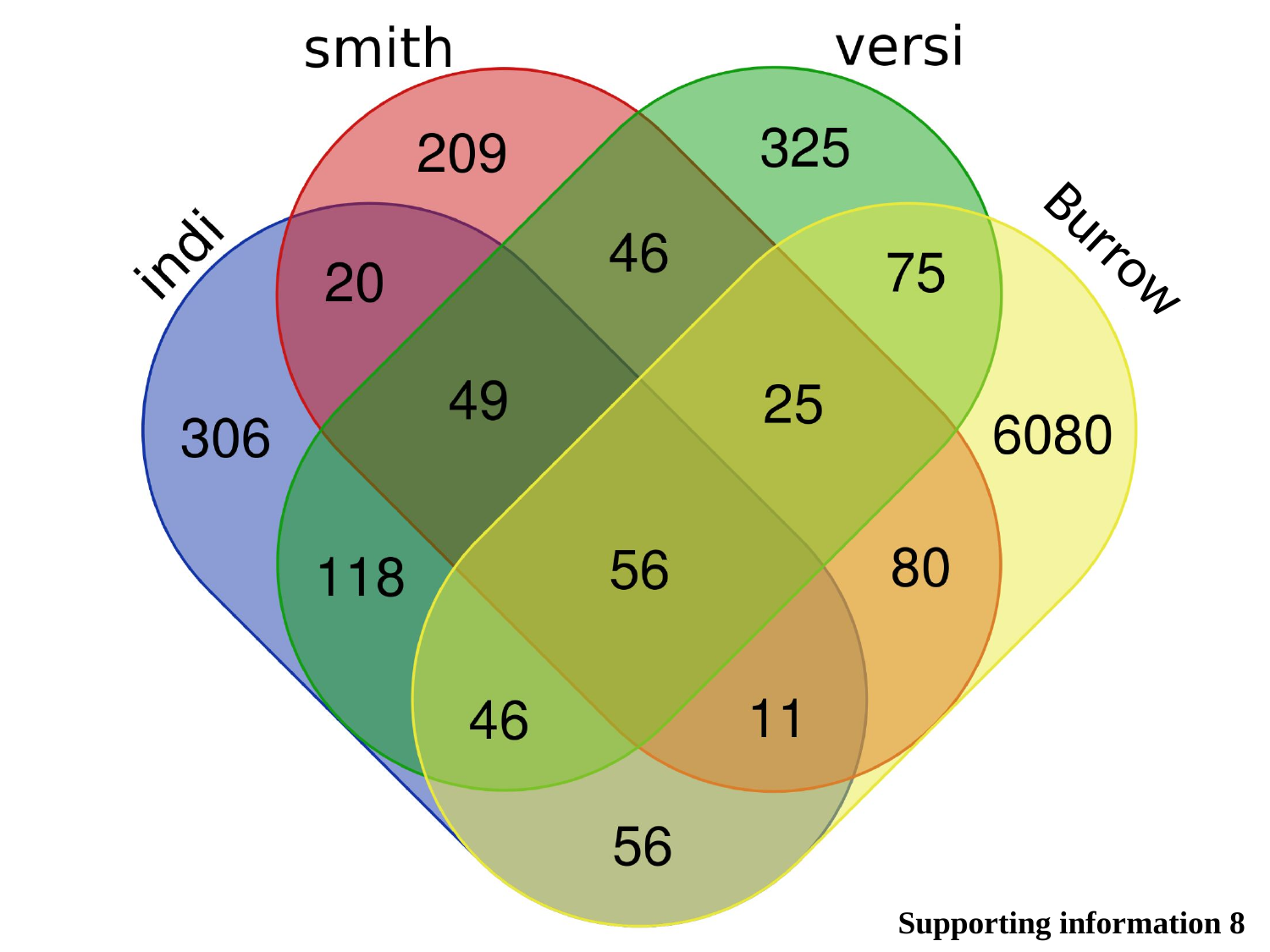

Burrow
Supporting information 8

Supplement: S8 File — (PPTX) [file pone.0261654.s008.pptx]
