# Supplementary material for: Mangrove crab intestine and habitat sediment microbiomes cooperatively work on carbon and nitrogen cycling
Source: PLoS One. 2021 Dec 31;16(12):e0261654. doi: 10.1371/journal.pone.0261654 (PMC8719709; doi:10.1371/journal.pone.0261654)
Supplement: S4 File — The bars represent the relative abundance of each sample. A) Crab intestine. B) Sediment region. (PPTX) [file pone.0261654.s004.pptx]

## Slide 1
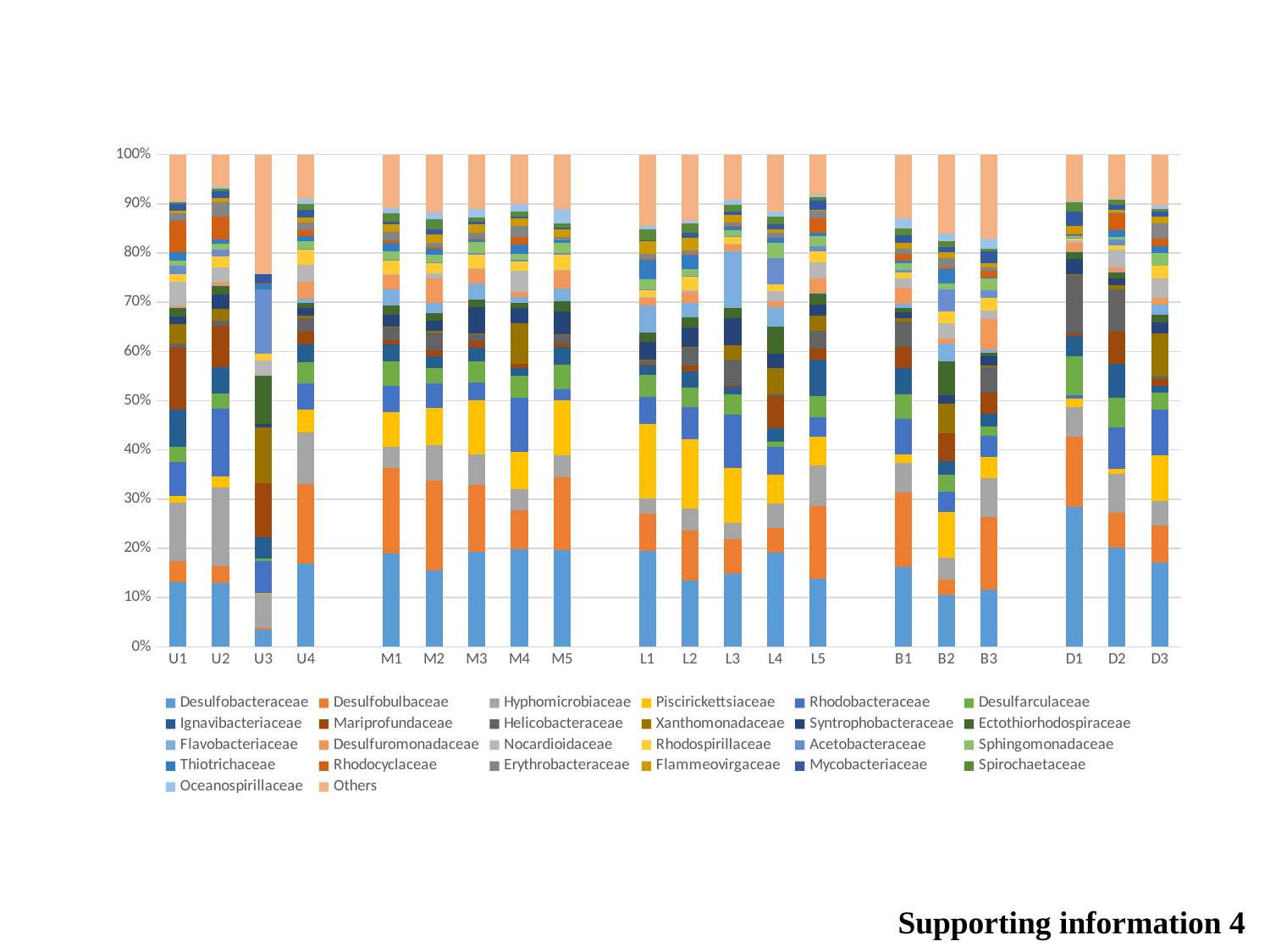

### Chart
| Category | Desulfobacteraceae | Desulfobulbaceae | Hyphomicrobiaceae | Piscirickettsiaceae | Rhodobacteraceae | Desulfarculaceae | Ignavibacteriaceae | Mariprofundaceae | Helicobacteraceae | Xanthomonadaceae | Syntrophobacteraceae | Ectothiorhodospiraceae | Flavobacteriaceae | Desulfuromonadaceae | Nocardioidaceae | Rhodospirillaceae | Acetobacteraceae | Sphingomonadaceae | Thiotrichaceae | Rhodocyclaceae | Erythrobacteraceae | Flammeovirgaceae | Mycobacteriaceae | Spirochaetaceae | Oceanospirillaceae | Others |
|---|---|---|---|---|---|---|---|---|---|---|---|---|---|---|---|---|---|---|---|---|---|---|---|---|---|---|
| U1 | 13.036906411386825 | 4.358231515304195 | 11.852878196246378 | 1.3225847084015618 | 6.890036528530041 | 3.2497795692152662 | 7.469454591258345 | 12.583448797077718 | 0.8691270940924549 | 3.9425620355208464 | 1.4485451568207584 | 1.8264265020783474 | 0.11336440357727673 | 0.4408615694671873 | 4.673132636352185 | 1.6500818742914725 | 1.6500818742914725 | 0.9950875425116513 | 1.7256581433429903 | 6.461771003904774 | 1.4737372465045975 | 0.6423982869379015 | 1.3477767980854012 | 0.28970903136415166 | 0.11336440357727673 | 9.572994079858901 |
| U2 | 12.928293889085129 | 3.4263511126810315 | 15.930766513599435 | 2.3313316849169903 | 13.811374072765807 | 2.9671494171670787 | 5.333804309431296 | 8.442246555987284 | 1.0596962204168139 | 2.472624514305899 | 2.8965030024726244 | 1.7308371600141292 | 0.10596962204168138 | 0.6358177322500883 | 3.037795831861533 | 2.1547156481808547 | 1.412928293889085 | 1.1656658424584951 | 0.9537265983751325 | 4.592016955139527 | 3.002472624514306 | 0.7417873542917697 | 1.4835747085835393 | 0.5298481102084069 | 0.2472624514305899 | 6.605439773931479 |
| U3 | 3.4445640473627557 | 0.43057050592034446 | 6.889128094725511 | 0.10764262648008611 | 6.458557588805167 | 0.6458557588805167 | 4.198062432723359 | 10.979547900968784 | 0.0 | 11.517761033369215 | 0.6458557588805167 | 9.68783638320775 | 0.0 | 0.0 | 3.1216361679224973 | 1.3993541442411195 | 13.02475780409042 | 0.0 | 1.0764262648008611 | 0.10764262648008611 | 0.0 | 0.0 | 1.9375672766415502 | 0.0 | 0.0 | 24.327233584499467 |
| U4 | 16.8944099378882 | 16.149068322981368 | 10.559006211180124 | 4.596273291925466 | 5.341614906832298 | 4.22360248447205 | 3.7267080745341614 | 2.484472049689441 | 2.857142857142857 | 0.4968944099378882 | 1.4906832298136645 | 0.9937888198757764 | 0.8695652173913043 | 3.4782608695652173 | 3.3540372670807455 | 3.1055900621118013 | 0.2484472049689441 | 1.6149068322981366 | 0.8695652173913043 | 1.3664596273291925 | 1.4906832298136645 | 0.9937888198757764 | 1.6149068322981366 | 1.1180124223602483 | 1.1180124223602483 | 8.944099378882015 |
| | None | None | None | None | None | None | None | None | None | None | None | None | None | None | None | None | None | None | None | None | None | None | None | None | None | None |
| M1 | 18.95945629247715 | 17.34239512538083 | 4.405905788610265 | 6.960393719240685 | 5.413639559409421 | 4.921490508554019 | 3.3981720178111083 | 0.8202484180923365 | 2.6482306069838293 | 0.0937426763534099 | 2.5076165924537146 | 1.8982891961565502 | 3.280993672369346 | 3.023201312397469 | 0.0937426763534099 | 2.7654089524255916 | 0.04687133817670495 | 1.8279821888914929 | 1.687368174361378 | 0.49214905085540195 | 1.687368174361378 | 1.6170611670963206 | 0.398406374501992 | 1.8045465198031403 | 0.9608624326224514 | 10.944457464260594 |
| M2 | 15.47111283443195 | 18.30166731291198 | 7.173322993408298 | 7.5610701822411785 | 4.963164017060876 | 3.1019775106630476 | 2.481582008530438 | 1.1244668476153548 | 3.722373012795657 | 0.23264831329972857 | 2.1326095385808452 | 1.589763474214812 | 1.9775106630476929 | 4.924389298177588 | 1.1632415664986429 | 2.016285381930981 | 0.15509887553315238 | 1.4734393175649476 | 1.3183404420317952 | 0.27142303218301667 | 0.9693679720822024 | 1.589763474214812 | 1.1632415664986429 | 2.055060100814269 | 1.240791004265219 | 11.826289259402856 |
| M3 | 19.312169312169313 | 13.624338624338625 | 6.084656084656085 | 11.054421768707483 | 3.684807256235828 | 4.270597127739985 | 2.6643990929705215 | 1.436130007558579 | 1.379440665154951 | 0.17006802721088435 | 5.347694633408919 | 1.4550264550264551 | 3.2879818594104306 | 3.0423280423280423 | 0.0944822373393802 | 2.7777777777777777 | 0.3779289493575208 | 2.1919879062736207 | 0.4346182917611489 | 0.15117157974300832 | 1.2093726379440666 | 1.7384731670445956 | 0.491307634164777 | 1.00151171579743 | 1.6439909297052153 | 11.073318216175352 |
| M4 | 19.81132075471698 | 7.861635220125786 | 4.40251572327044 | 7.547169811320755 | 11.0062893081761 | 4.40251572327044 | 1.5723270440251573 | 0.9433962264150944 | 0.0 | 8.176100628930818 | 3.1446540880503147 | 0.9433962264150944 | 1.2578616352201257 | 0.9433962264150944 | 4.40251572327044 | 1.8867924528301887 | 0.31446540880503143 | 1.2578616352201257 | 1.8867924528301887 | 1.5723270440251573 | 2.20125786163522 | 1.5723270440251573 | 0.31446540880503143 | 0.9433962264150944 | 1.5723270440251573 | 10.062893081761004 |
| M5 | 19.573901464713714 | 14.824678206835332 | 4.571682201509099 | 11.051930758988016 | 2.3080337328007103 | 5.015534842432312 | 3.5952063914780292 | 0.6657789613848203 | 1.6866400355082112 | 0.13315579227696406 | 4.749223257878384 | 2.0861074123391035 | 2.5299600532623168 | 3.683976919662672 | 0.0887705281846427 | 3.1069684864624945 | 0.31069684864624947 | 2.130492676431425 | 0.3994673768308921 | 0.0 | 0.7545494895694629 | 1.4647137150466045 | 0.3994673768308921 | 0.8433200177541056 | 2.8850421660008876 | 11.14070128717269 |
| | None | None | None | None | None | None | None | None | None | None | None | None | None | None | None | None | None | None | None | None | None | None | None | None | None | None |
| L1 | 19.41919191919192 | 7.6767676767676765 | 3.0555555555555554 | 15.126262626262626 | 5.454545454545454 | 4.570707070707071 | 1.792929292929293 | 0.20202020202020202 | 0.9595959595959596 | 0.050505050505050504 | 3.5606060606060606 | 2.0454545454545454 | 5.505050505050505 | 1.5151515151515151 | 0.15151515151515152 | 1.5151515151515151 | 0.07575757575757576 | 1.994949494949495 | 4.015151515151516 | 0.050505050505050504 | 1.0606060606060606 | 2.7525252525252526 | 0.025252525252525252 | 2.196969696969697 | 0.7575757575757576 | 14.469696969696955 |
| L2 | 13.482142857142858 | 10.178571428571429 | 4.419642857142857 | 14.151785714285714 | 6.473214285714286 | 3.8839285714285716 | 3.3482142857142856 | 1.1160714285714286 | 3.75 | 0.044642857142857144 | 3.9732142857142856 | 2.0535714285714284 | 2.9464285714285716 | 2.2767857142857144 | 0.26785714285714285 | 2.767857142857143 | 0.17857142857142858 | 1.3839285714285714 | 2.9464285714285716 | 0.08928571428571429 | 0.8482142857142857 | 2.5 | 0.9821428571428571 | 1.9196428571428572 | 0.625 | 13.392857142857153 |
| L3 | 14.761092150170649 | 7.081911262798635 | 3.2849829351535837 | 11.220136518771332 | 10.83617747440273 | 4.138225255972697 | 1.4789533560864618 | 0.31285551763367464 | 5.190557451649602 | 2.915244596131968 | 5.546075085324232 | 2.033560864618885 | 11.532992036405005 | 1.422070534698521 | 0.19908987485779295 | 1.350967007963595 | 0.17064846416382254 | 1.2087599544937428 | 0.7821387940841866 | 0.02844141069397042 | 0.7536973833902162 | 1.5216154721274175 | 0.6683731513083049 | 1.350967007963595 | 1.1234357224118316 | 9.087030716723547 |
| L4 | 19.106699751861044 | 4.962779156327543 | 4.962779156327543 | 5.955334987593052 | 5.707196029776675 | 0.9925558312655087 | 2.729528535980149 | 6.451612903225806 | 0.49627791563275436 | 5.2109181141439205 | 2.977667493796526 | 5.459057071960298 | 3.970223325062035 | 1.2406947890818858 | 1.9851116625310175 | 1.488833746898263 | 5.2109181141439205 | 3.225806451612903 | 0.9925558312655087 | 0.0 | 0.9925558312655087 | 0.7444168734491315 | 0.9925558312655087 | 1.488833746898263 | 0.9925558312655087 | 11.662531017369744 |
| L5 | 13.86430678 | 14.74926254 | 8.259587021 | 5.752212389 | 3.982300885 | 4.277286136 | 7.522123894 | 2.212389381 | 3.539823009 | 3.097345133 | 2.212389381 | 2.359882006 | 0.147492625 | 2.802359882 | 3.392330383 | 2.212389381 | 1.032448378 | 2.064896755 | 0.589970501 | 2.949852507 | 1.622418879 | 0.147492625 | 1.91740413 | 0.589970501 | 0.442477876 | 8.259587022000005 |
| | None | None | None | None | None | None | None | None | None | None | None | None | None | None | None | None | None | None | None | None | None | None | None | None | None | None |
| B1 | 16.13466836906122 | 15.200591358107655 | 6.0681405819501375 | 1.6262347960486527 | 7.338216517707143 | 5.0063839795712655 | 5.187823398965123 | 4.394865936429003 | 5.187823398965123 | 0.6585578926147436 | 1.2499160002687992 | 0.8534372690007392 | 0.7593575700557759 | 3.2524695920973055 | 1.8412741079228547 | 1.2297560647805927 | 0.645117935622606 | 1.2364760432766615 | 0.5913581076540555 | 1.4044755056783818 | 1.041596666890666 | 1.2095961292923862 | 1.4985552046233452 | 1.3708756131980377 | 2.083193333781332 | 12.929238626436387 |
| B2 | 10.597302504816955 | 3.0828516377649327 | 4.431599229287091 | 9.248554913294798 | 4.238921001926783 | 3.275529865125241 | 2.8901734104046244 | 5.5876685934489405 | 0.0 | 5.973025048169557 | 1.7341040462427746 | 6.936416184971098 | 3.6608863198458574 | 0.9633911368015414 | 3.0828516377649327 | 2.504816955684008 | 4.431599229287091 | 1.1560693641618498 | 2.8901734104046244 | 0.3853564547206166 | 1.9267822736030829 | 1.1560693641618498 | 0.9633911368015414 | 1.348747591522158 | 1.5414258188824663 | 15.992292870905572 |
| B3 | 11.608300907911802 | 14.785992217898833 | 7.911802853437095 | 4.345006485084306 | 4.280155642023346 | 1.880674448767834 | 2.4643320363164722 | 4.409857328145266 | 5.058365758754864 | 0.45395590142671854 | 1.7509727626459144 | 0.7133592736705577 | 0.7782101167315175 | 6.22568093385214 | 1.6212710765239948 | 2.594033722438392 | 1.6212710765239948 | 2.2697795071335927 | 0.324254215304799 | 1.2321660181582361 | 0.7782101167315175 | 0.9079118028534371 | 2.2697795071335927 | 0.5836575875486382 | 2.0752269779507135 | 17.055771725032415 |
| | None | None | None | None | None | None | None | None | None | None | None | None | None | None | None | None | None | None | None | None | None | None | None | None | None | None |
| D1 | 28.49702380952381 | 14.16170634920635 | 6.063988095238095 | 1.773313492063492 | 0.6696428571428571 | 7.862103174603175 | 4.141865079365079 | 0.533234126984127 | 11.941964285714286 | 0.0496031746031746 | 3.013392857142857 | 1.3764880952380953 | 0.1984126984126984 | 1.7113095238095237 | 0.5208333333333334 | 0.44642857142857145 | 0.1984126984126984 | 0.2728174603174603 | 0.16121031746031747 | 0.1984126984126984 | 0.11160714285714286 | 1.6741071428571428 | 2.8273809523809526 | 1.8973214285714286 | 0.21081349206349206 | 9.486607142857167 |
| D2 | 20.11888431641518 | 7.087334247828075 | 7.9103795153177865 | 1.005944215820759 | 8.50480109739369 | 5.94421582075903 | 6.950160036579789 | 6.44718792866941 | 8.596250571559214 | 0.9602194787379973 | 1.2802926383173296 | 1.417466849565615 | 0.04572473708276177 | 0.823045267489712 | 3.5665294924554183 | 0.9144947416552355 | 1.2345679012345678 | 0.411522633744856 | 1.417466849565615 | 3.5208047553726565 | 0.09144947416552354 | 0.5486968449931413 | 0.9144947416552355 | 1.0516689529035208 | 0.22862368541380887 | 9.007773205304062 |
| D3 | 17.01795472 | 7.650273224 | 4.918032787 | 9.367681499 | 9.289617486 | 3.43481655 | 1.405152225 | 1.327088212 | 0.468384075 | 8.821233411 | 2.263856362 | 1.405152225 | 2.18579235 | 1.327088212 | 3.981264637 | 2.576112412 | 0.0 | 2.576112412 | 1.405152225 | 1.56128025 | 2.966432475 | 1.405152225 | 1.014832162 | 0.6245121 | 0.702576112 | 10.304449652000002 |Supporting information 4

## Slide 2
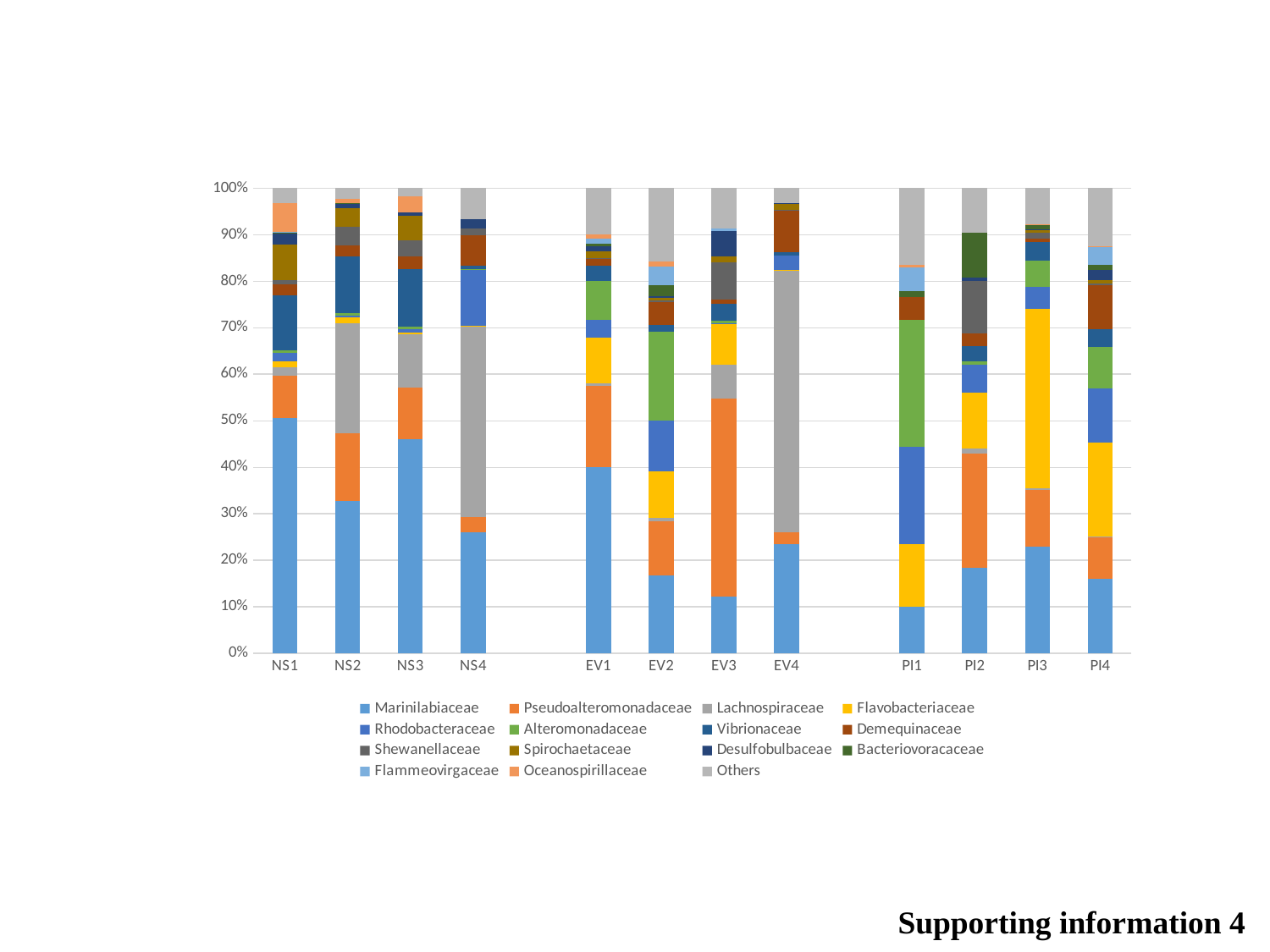

### Chart
| Category | Marinilabiaceae | Pseudoalteromonadaceae | Lachnospiraceae | Flavobacteriaceae | Rhodobacteraceae | Alteromonadaceae | Vibrionaceae | Demequinaceae | Shewanellaceae | Spirochaetaceae | Desulfobulbaceae | Bacteriovoracaceae | Flammeovirgaceae | Oceanospirillaceae | Others |
|---|---|---|---|---|---|---|---|---|---|---|---|---|---|---|---|
| NS1 | 50.63291139240506 | 9.124472573839663 | 1.7932489451476794 | 1.160337552742616 | 1.8459915611814346 | 0.5274261603375527 | 11.814345991561181 | 2.3734177215189876 | 0.9493670886075949 | 7.70042194092827 | 2.478902953586498 | 0.10548523206751055 | 0.052742616033755275 | 6.276371308016878 | 3.1645569620253013 |
| NS2 | 32.836710369487484 | 14.451728247914184 | 23.71871275327771 | 1.162097735399285 | 0.4171632896305125 | 0.4767580452920143 | 12.336114421930871 | 2.3539928486293205 | 4.022646007151371 | 3.843861740166865 | 1.0131108462455305 | 0.23837902264600716 | 0.0 | 0.7449344457687723 | 2.3837902264600785 |
| NS3 | 46.02564102564103 | 11.153846153846153 | 11.41025641025641 | 0.38461538461538464 | 0.7692307692307693 | 0.5128205128205128 | 12.435897435897436 | 2.6923076923076925 | 3.4615384615384617 | 5.256410256410256 | 0.7692307692307693 | 0.0 | 0.0 | 3.3333333333333335 | 1.7948717948717956 |
| NS4 | 26.00502512562814 | 3.391959798994975 | 40.95477386934673 | 0.12562814070351758 | 11.93467336683417 | 0.12562814070351758 | 0.8793969849246231 | 6.532663316582915 | 1.3819095477386936 | 0.0 | 2.0100502512562812 | 0.0 | 0.0 | 0.0 | 6.658291457286438 |
| | None | None | None | None | None | None | None | None | None | None | None | None | None | None | None |
| EV1 | 40.03389830508475 | 17.52542372881356 | 0.5423728813559322 | 9.728813559322035 | 3.7966101694915255 | 8.508474576271187 | 3.2203389830508473 | 1.4915254237288136 | 0.13559322033898305 | 1.3898305084745763 | 1.0508474576271187 | 0.6440677966101694 | 1.0847457627118644 | 0.9152542372881356 | 9.932203389830491 |
| EV2 | 16.791979949874687 | 11.528822055137844 | 0.7518796992481203 | 10.025062656641603 | 11.027568922305765 | 19.047619047619047 | 1.5037593984962405 | 4.761904761904762 | 0.5012531328320802 | 0.5012531328320802 | 0.2506265664160401 | 2.506265664160401 | 4.010025062656641 | 1.0025062656641603 | 15.789473684210535 |
| EV3 | 12.27154046997389 | 42.55874673629243 | 7.310704960835509 | 8.616187989556137 | 0.26109660574412535 | 0.5221932114882507 | 3.6553524804177546 | 0.783289817232376 | 8.093994778067884 | 1.3054830287206267 | 5.483028720626632 | 0.0 | 0.5221932114882507 | 0.0 | 8.61618798955611 |
| EV4 | 23.406040268456376 | 2.6006711409395975 | 56.375838926174495 | 0.08389261744966443 | 3.0201342281879193 | 0.0 | 0.7550335570469798 | 8.89261744966443 | 0.2516778523489933 | 1.174496644295302 | 0.16778523489932887 | 0.0 | 0.0 | 0.0 | 3.271812080536918 |
| | None | None | None | None | None | None | None | None | None | None | None | None | None | None | None |
| PI1 | 10.0 | 0.0 | 0.0 | 13.478260869565217 | 20.869565217391305 | 27.391304347826086 | 0.0 | 4.782608695652174 | 0.0 | 0.0 | 0.0 | 1.3043478260869565 | 5.217391304347826 | 0.43478260869565216 | 16.52173913043478 |
| PI2 | 18.306010928961747 | 24.59016393442623 | 1.092896174863388 | 12.021857923497267 | 6.0109289617486334 | 0.819672131147541 | 3.278688524590164 | 2.73224043715847 | 11.202185792349727 | 0.0 | 0.819672131147541 | 9.562841530054644 | 0.0 | 0.0 | 9.56284153005467 |
| PI3 | 22.872702518720217 | 12.253233492171546 | 0.27229407760381213 | 38.66575901974132 | 4.765146358066712 | 5.582028590878148 | 4.016337644656229 | 0.8168822328114363 | 1.2253233492171545 | 0.6807351940095303 | 0.06807351940095303 | 0.9530292716133424 | 0.0 | 0.06807351940095303 | 7.7603812117086335 |
| PI4 | 16.04859335038363 | 8.951406649616368 | 0.1918158567774936 | 20.076726342710998 | 11.70076726342711 | 8.823529411764707 | 3.9002557544757033 | 9.526854219948849 | 0.2557544757033248 | 0.7033248081841432 | 2.3017902813299234 | 0.959079283887468 | 4.028132992327365 | 0.1278772378516624 | 12.404092071611245 |Supporting information 4
