# Supplementary material for: Mangrove crab intestine and habitat sediment microbiomes cooperatively work on carbon and nitrogen cycling
Source: PLoS One. 2021 Dec 31;16(12):e0261654. doi: 10.1371/journal.pone.0261654 (PMC8719709; doi:10.1371/journal.pone.0261654)
Supplement: S6 File — (PPTX) [file pone.0261654.s006.pptx]

## Slide 1
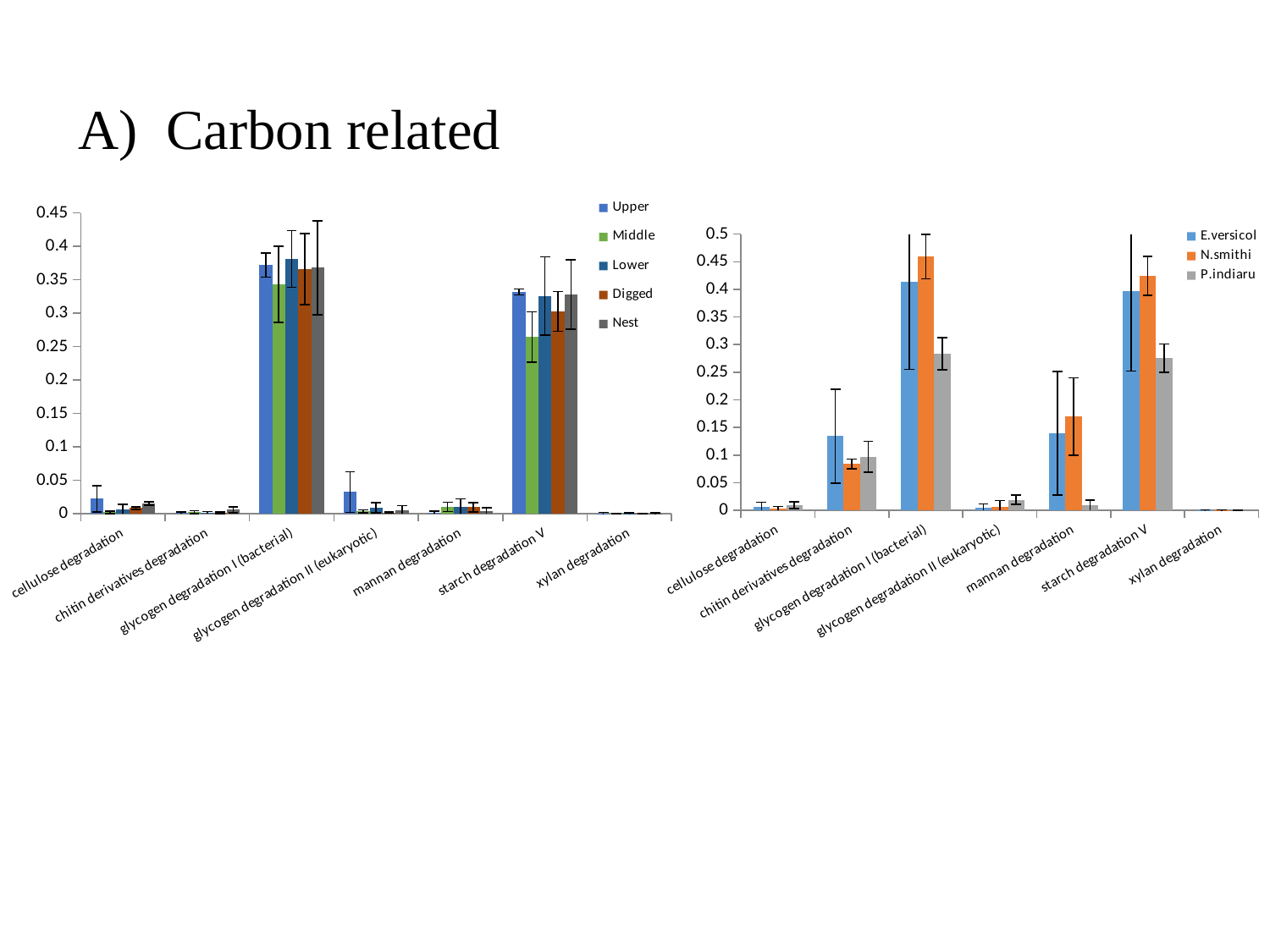

A) Carbon related
### Chart
| Category | Upper | Middle | Lower | Digged | Nest |
|---|---|---|---|---|---|
| cellulose degradation | 0.0226395695108 | 0.00234895465351 | 0.00644481157567 | 0.00852885617976 | 0.0156351987646 |
| chitin derivatives degradation | 0.00102566284455 | 0.00255603305323 | 0.00158545222745 | 0.00179425307038 | 0.00602926361463 |
| glycogen degradation I (bacterial) | 0.371866959728 | 0.343213474401 | 0.381280771852 | 0.365846537568 | 0.368020952256 |
| glycogen degradation II (eukaryotic) | 0.0326394368558 | 0.00364219621433 | 0.00895704055492 | 0.00174890704389 | 0.00510760247558 |
| mannan degradation | 0.00174802945945 | 0.0102705483174 | 0.00991020027979 | 0.00990625737067 | 0.00369434808635 |
| starch degradation V | 0.331815886472 | 0.264480747716 | 0.325660200665 | 0.302589766375 | 0.32797156217 |
| xylan degradation | 0.000819621751735 | 0.000175096175953 | 0.000681969747247 | 0.000500453551556 | 0.000612712124745 |
### Chart
| Category | E.versicol | N.smithi | P.indiaru |
|---|---|---|---|
| cellulose degradation | 0.00651689006056 | 0.00311586251318 | 0.00934280690638 |
| chitin derivatives degradation | 0.134397389319 | 0.0839392918585 | 0.0969871564726 |
| glycogen degradation I (bacterial) | 0.41374661787 | 0.459449801788 | 0.283509581148 |
| glycogen degradation II (eukaryotic) | 0.00545398375723 | 0.006406821402 | 0.0192801823205 |
| mannan degradation | 0.139406993216 | 0.169731059097 | 0.00918347876764 |
| starch degradation V | 0.397027121348 | 0.424364610964 | 0.275470828693 |
| xylan degradation | 0.000283826458337 | 0.000259325025359 | 0.0 |

## Slide 2
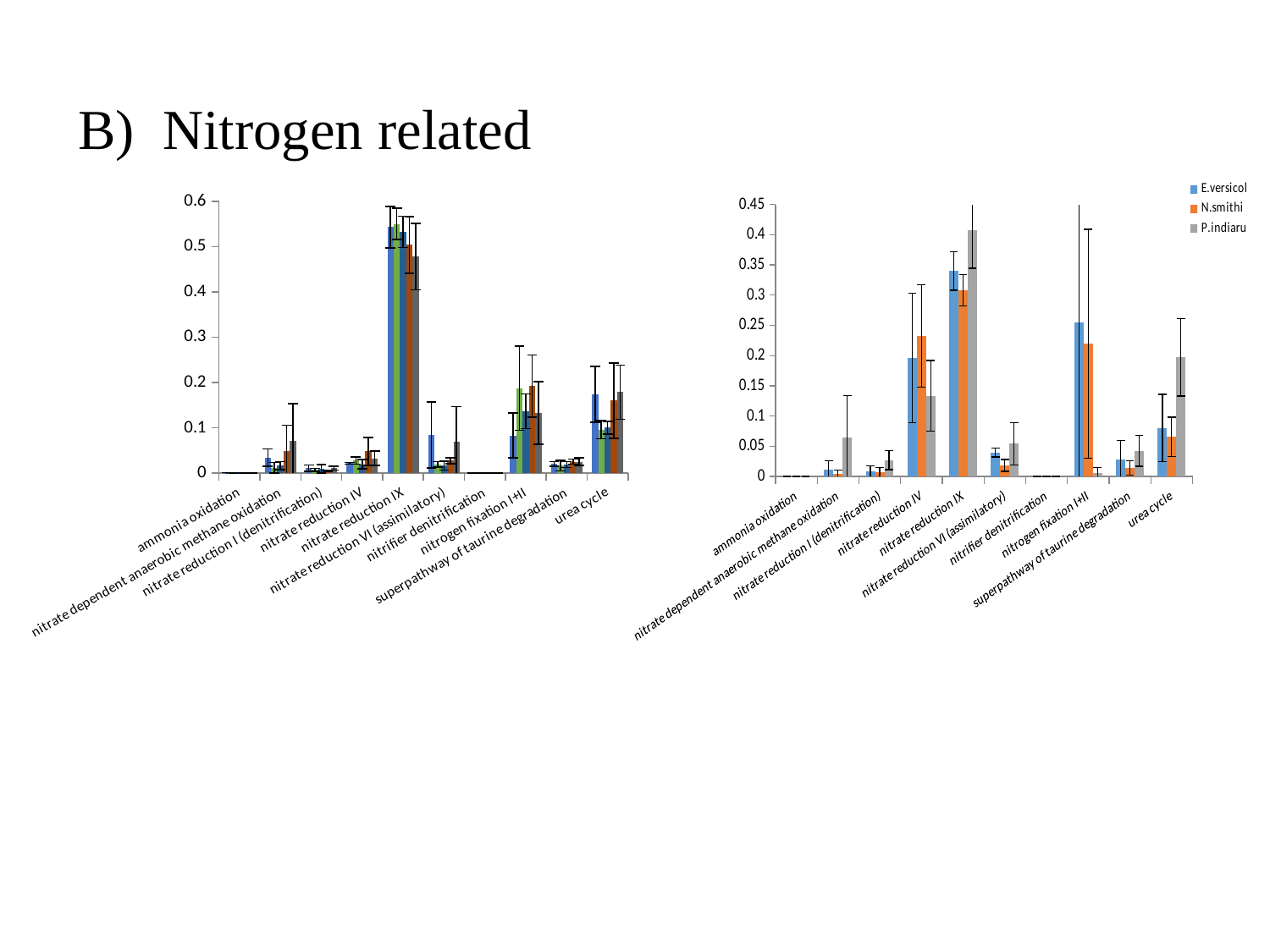

B) Nitrogen related
### Chart
| Category | E.versicol | N.smithi | P.indiaru |
|---|---|---|---|
| ammonia oxidation | 0.0 | 0.0 | 0.0 |
| nitrate dependent anaerobic methane oxidation | 0.010822358803 | 0.00488221965692 | 0.0639951855861 |
| nitrate reduction I (denitrification) | 0.00828228224588 | 0.00694254631161 | 0.0271869832555 |
| nitrate reduction IV | 0.196083527363 | 0.232567536081 | 0.133482939292 |
| nitrate reduction IX | 0.34004182761 | 0.308134727228 | 0.408211489839 |
| nitrate reduction VI (assimilatory) | 0.0399151768144 | 0.0182379449469 | 0.0542568214359 |
| nitrifier denitrification | 0.0 | 0.0 | 0.0 |
| nitrogen fixation I+II | 0.255506921185 | 0.219660474263 | 0.00624585453435 |
| superpathway of taurine degradation | 0.0287679614219 | 0.0144123675763 | 0.0423983316198 |
| urea cycle | 0.0804129070347 | 0.065684538068 | 0.197138867541 |
### Chart
| Category | Upper | Middle | Lower | Digged | Nest |
|---|---|---|---|---|---|
| ammonia oxidation | 0.000552488389207 | 0.000883219095764 | 0.000744234255516 | 0.000541557843038 | 0.00100048482359 |
| nitrate dependent anaerobic methane oxidation | 0.0346185656081 | 0.0120719811262 | 0.0164708640301 | 0.0494491469015 | 0.0708656582889 |
| nitrate reduction I (denitrification) | 0.0113430213651 | 0.0073616316486 | 0.00959404646932 | 0.00550483497042 | 0.0110362664516 |
| nitrate reduction IV | 0.0220008754094 | 0.027913883298 | 0.0200251521361 | 0.0481217608502 | 0.0330221451341 |
| nitrate reduction IX | 0.543260060223 | 0.550115008382 | 0.532699267187 | 0.503595574832 | 0.477764577105 |
| nitrate reduction VI (assimilatory) | 0.0845044815823 | 0.0191745983792 | 0.0169784129869 | 0.0274609495425 | 0.0693270906676 |
| nitrifier denitrification | 0.0 | 0.0 | 0.000174807349709 | 0.0 | 0.000108306892192 |
| nitrogen fixation I+II | 0.0832792784056 | 0.187324716256 | 0.13662320825 | 0.192381112611 | 0.133083420902 |
| superpathway of taurine degradation | 0.0204661836503 | 0.0167175367234 | 0.0187675364637 | 0.0254319210407 | 0.0252972651214 |
| urea cycle | 0.174243354959 | 0.0962130746849 | 0.100480551262 | 0.160214281098 | 0.17885586778 |

## Slide 3
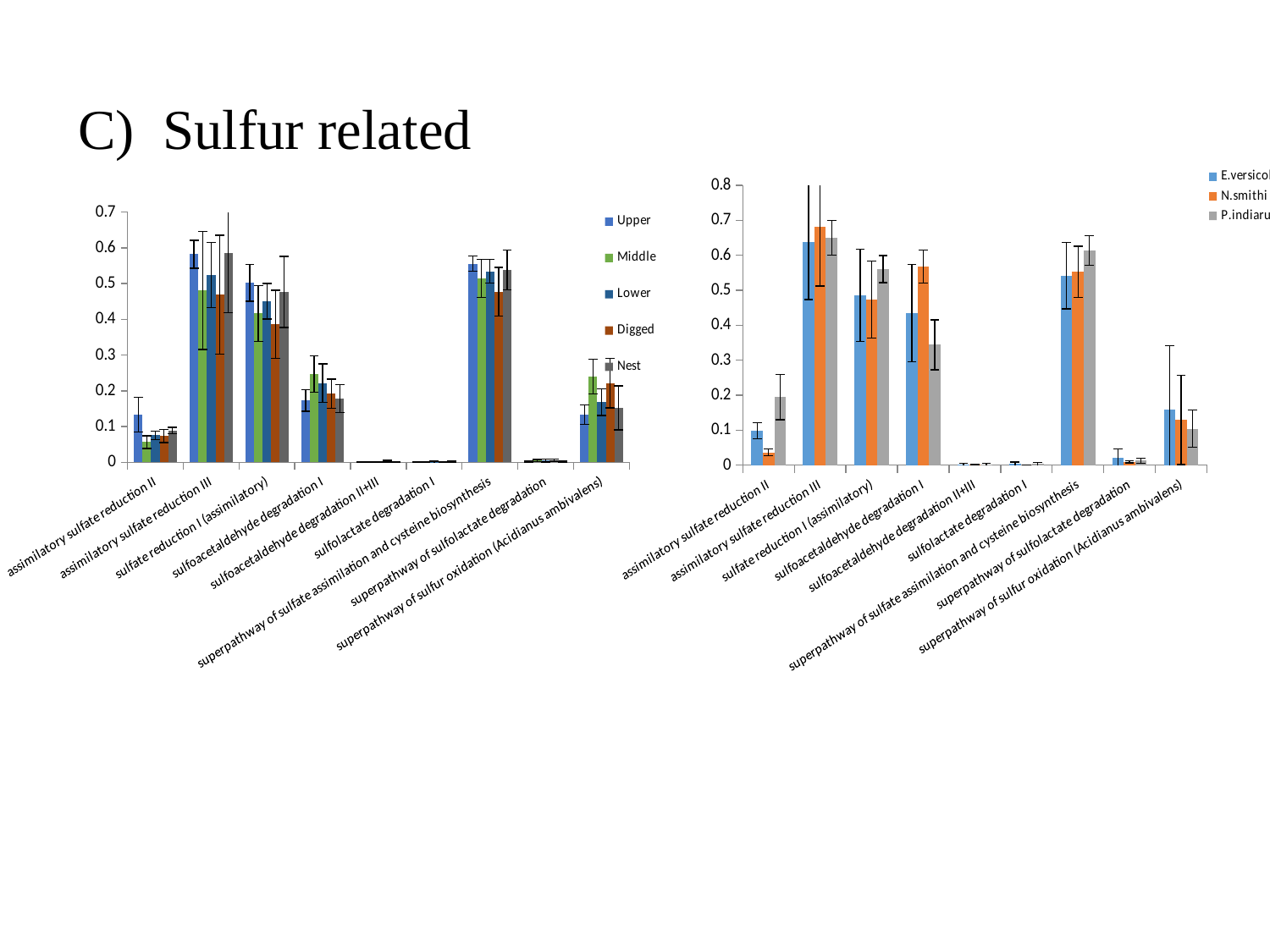

C) Sulfur related
### Chart
| Category | E.versicol | N.smithi | P.indiaru |
|---|---|---|---|
| assimilatory sulfate reduction II | 0.0981801416333 | 0.0366726802473 | 0.194767933389 |
| assimilatory sulfate reduction III | 0.638509169224 | 0.68181818509 | 0.650328965747 |
| sulfate reduction I (assimilatory) | 0.485852240993 | 0.47364950735 | 0.560725348794 |
| sulfoacetaldehyde degradation I | 0.434891094796 | 0.568240425979 | 0.344259511203 |
| sulfoacetaldehyde degradation II+III | 0.00216675341131 | 0.0013214154956 | 0.00206928821312 |
| sulfolactate degradation I | 0.0034106924695 | 0.0 | 0.0035015004036 |
| superpathway of sulfate assimilation and cysteine biosynthesis | 0.541636760793 | 0.552402037922 | 0.613806382236 |
| superpathway of sulfolactate degradation | 0.021751258914 | 0.00946518628207 | 0.0130098910738 |
| superpathway of sulfur oxidation (Acidianus ambivalens) | 0.158221797545 | 0.129377742268 | 0.10437642873 |
### Chart
| Category | Upper | Middle | Lower | Digged | Nest |
|---|---|---|---|---|---|
| assimilatory sulfate reduction II | 0.133255426651 | 0.0563301797735 | 0.0751755388327 | 0.0732840965825 | 0.0891407119421 |
| assimilatory sulfate reduction III | 0.582258194573 | 0.480547214401 | 0.523929816428 | 0.469042954896 | 0.584718720126 |
| sulfate reduction I (assimilatory) | 0.501998229858 | 0.416455589381 | 0.450661472686 | 0.386084400766 | 0.476467656965 |
| sulfoacetaldehyde degradation I | 0.17327319246 | 0.246851059506 | 0.221793115615 | 0.191719017234 | 0.178225204722 |
| sulfoacetaldehyde degradation II+III | 0.000427037142775 | 0.00107450144616 | 0.000362136342665 | 0.00296834699431 | 0.000693087574861 |
| sulfolactate degradation I | 0.0012240556374 | 0.0013149365986 | 0.0012688112492 | 0.000387539704982 | 0.00158131856873 |
| superpathway of sulfate assimilation and cysteine biosynthesis | 0.556005393745 | 0.51480909103 | 0.534554641853 | 0.477364077596 | 0.53823504223 |
| superpathway of sulfolactate degradation | 0.00185592823851 | 0.00448208471099 | 0.00463121610669 | 0.00573316614705 | 0.00156951415356 |
| superpathway of sulfur oxidation (Acidianus ambivalens) | 0.132566682906 | 0.239377701788 | 0.168400801651 | 0.221672476638 | 0.152064972608 |
